# Blurring evidence with advocacy: a systematic review of policy recommendations for net zero

**DOI:** 10.1038/s44432-026-00012-6

**Published:** 2026-06-09

**Authors:** Evangelos Danopoulos, Aarushi Shah, Claudia R. Schneider, John A. D. Aston

**Affiliations:** 1 Statistical Laboratory, Department of Pure Mathematics and Mathematical Statistics, University of Cambridge, Cambridge, United Kingdom; 2 School of Psychology, Speech and Hearing, University of Canterbury, Christchurch, New Zealand

**Keywords:** Climate change, Energy policy, Climate-change mitigation, Climate-change policy, Environmental sciences, Environmental social sciences, Environmental studies

## Abstract

Policy recommendations (PRs) now play a crucial role in tackling the climate crisis, underscoring the need for evidence-based, objective and transparent communication. Scientific studies often put forward PRs, but their quality has not yet been evaluated. Here, we systematically appraised the quality of PRs in areas of green energy and transportation for tackling climate change. Four databases were searched from 2019. Over three thousand papers were considered, and 23 studies are included. Quality rating, based on the Evidence Communication Rules for Policy (ECR-P) critical appraisal tool, indicated poor quality in PRs across all studies. There was a clear trend towards advocacy as opposed to providing neutral information, further exacerbated by inadequate disclosure of uncertainties. Communication quality was markedly better regarding study findings and conclusions compared to PRs. Researchers should use the same scientific rigour and reporting standards in PRs as in any other aspect of their research. A reporting guideline for scientific-based PRs could greatly assist in this area, elevating the standards of communication for both academic publishing and global policymaking while reducing potential criticism that scientists simply put forward their own opinions.

## Introduction

Scientific evidence is continually being produced and disseminated, with peer-reviewed journals being the main source of distribution. Indeed, the number of new papers published every day, even within a single discipline, is often prohibitive for non-experts^1^. Evidence is often presented in a manner easily recognisable to members of the same field but inaccessible to ‘outsiders’. Although acceptable within the scientific community, this creates barriers to knowledge mobilisation beyond academia and even between disciplines^1^. Furthermore, the quality of evidence communication in scientific papers will undoubtedly affect accessibility, comprehension and use in decision making^2^.

Following initial successful application in healthcare, the use of scientific evidence to underpin public policies has been gaining increasing support and is now expanding in other policy areas as well^3^. As such, evidence communication targeted at policy makers can be seen as part of knowledge mobilisation and part of primary research dissemination in peer-reviewed papers^4^.

Evaluation of evidence communication requires conceptualisation of knowledge, knowledge exchange and their intended outcomes^5^. Evidence-based policymaking follows a versatile route and depends on a wide range of factors and actors beyond the narrow scientific dimension - this inherent complexity is only partially captured by existing conceptualisation models^6,7^. In practice, although scientific evidence is central to policy development, its use depends on the timely accessibility of high-quality research^1^. Despite the acknowledged importance of evidence in this context, what constitutes good evidence communication remains under-specified.

This gap highlights the need for a framework that distinguishes the communication of evidence from advocacy and provides guidance suited to the complexities of policymaking. The five rules for evidence communication developed by the Winton Centre for Risk and Evidence Communication address this need^2^. This approach proposes that when scientists communicate evidence, they should: inform, not persuade (refrain from advocacy); offer balance, but not false balance; disclose uncertainties; state evidence quality, and inoculate against misinformation (pre-empt misunderstandings). The rules offer a theoretically grounded approach for good practice in evidence communication. The majority of scientific research around evidence-based policymaking has so far focused on healthcare and public health policy^8^. In the present work, the focus is on environment-related policies.

Mitigating climate change and the race to reach net zero is gaining increasingly more attention in evidence-based policy settings^9^. A significant amount of scientific evidence in this area is continually being produced, contributing to policymaking debates and high-stakes decisions. While evidence is urgently needed to support policies, this pressure might push scientists to blur the line between objective analysis and engaging in advocacy. Therefore, understanding how evidence for policies is communicated in this context is crucial.

The focus of this systematic review was scientific papers which communicate policy recommendations within the environmental domain. The objectives of the systematic review were to examine how authors communicate scientific evidence in support of their policy recommendations (PRs) in the areas of green energy and transportation for net zero, and climate change mitigation; and to examine what is the quality of scientific-based PRs in the same areas. Specifically, green energy here means wind power and hydrogen energy, while transportation relates to road vehicles. We have examined these prominent areas of policy innovation around the green agenda as exemplars of ideas in net zero. These areas were highlighted in the recent *Net Zero Strategy: Build Back Greener* policy of the UK government^9^ as key areas for reducing emissions across the economy.

## Results

### Characteristics of the evaluated literature

**Fig. 1:**
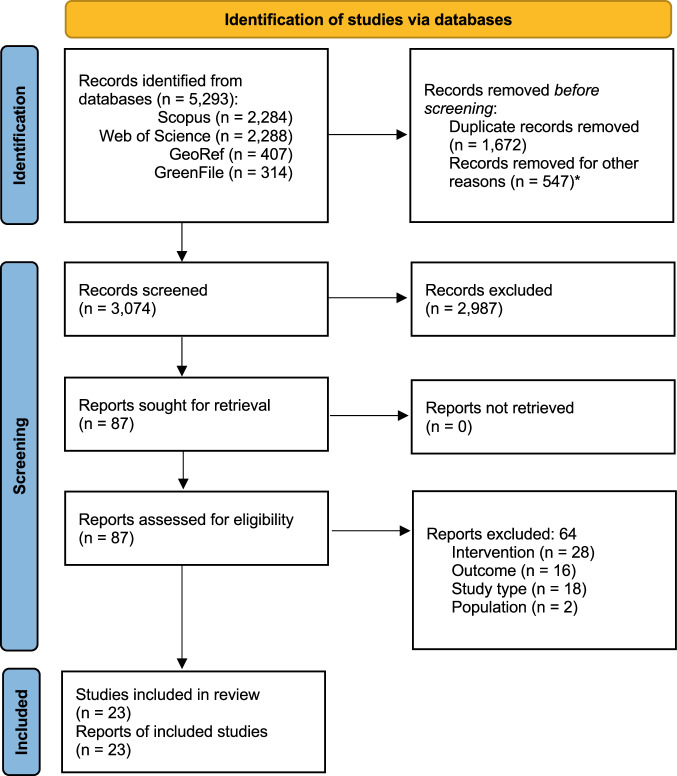
PRISMA flow diagram. *Records of books (*n* = 268), conference proceedings (*n* = 179), and theses (*n* = 100) were removed before screening as they are beyond the focus of this systematic review. The screening process is reported in a PRISMA (Preferred Reporting Items for Systematic Reviews and Meta-Analyses) flow diagram.

The search strategies in the four bibliographic databases identified 5293 records, which were reduced to 3621 unique records after de-duplication. In addition, 547 further records were excluded as their type was beyond the scope of this systematic review, thus leaving 3074 records for the first stage of screening. During screening of titles and abstracts, 87 records met the eligibility criteria and were carried over to the second stage of full paper screening. Twenty-three studies^10–32^ were ultimately included in the systematic review after the conclusion of the full paper screening. The selection process, including the reasons for exclusion during full paper screening, is illustrated in a PRISMA flowchart in Fig. 1. A list of the excluded studies along with the reason for exclusion can be found in Supplementary Table 1.

Details of the included studies are presented in Table 1. Out of the 23 studies, 18^12–29^ included wind power as an intervention in tackling climate change or reaching the net zero target (decarbonisation), while one focused on hydrogen^32^ and one on transportation^30^. Three studies focused on more than one of the themes of this systematic review. One study^10^ looked at both wind power and transportation, one^11^ at both wind power and hydrogen energy and one^31^ at both hydrogen energy and transportation.

**Table 1:** Study details

Study	Area of research	Methodology	Methods	Data source (for models)	Research country of origin	Research country focus	Intervention
Jahanger et al.^14^	Wind power	Econometric/ empirical modelling	MMQR; DK-SE	WDI	China, United Arab Emirates, Turkey, Taiwan, Nigeria, Australia, Bangladesh	10 countries^a^	Technology and renewable energy impact on energy efficiency and carbon neutrality
Zhao, C. et al.^15^	Wind power	Econometric/ empirical modelling	IV-GMM	WDI, BP	China	77 countries^b^	Renewable energy use for carbon unlocking
Raihan et al.^16^	Wind power	Econometric/ empirical modelling	ARDL; DOLS	WDI	Malaysia	Thailand	Dynamic effects of economic growth, renewable energy use, urbanisation, industrialisation, tourism, agricultural productivity, and forest area on CO^2^ emissions in Thailand.
Hossain et al.^17^	Wind power	Econometric/ empirical modelling	DARDL; ARDL; FMOLS	BP, OECD, WDI, peer-reviewed paper	Bangladesh, China, Australia, USA	USA	Eco-innovation, nuclear energy consumption, fossil fuel consumption, and renewable energy consumption impact on Load Capacity Factor
Raihan and Tuspekova^18^	Wind power	Econometric/ empirical modelling	DOLS	WDI	Malaysia	Turkey	Dynamic effects of economic growth, renewable energy use, urbanisation, industrialisation, tourism, agricultural productivity, and forest area on CO^2^ emissions in Turkey.
Raihan et al.^19^	Wind power	Econometric/ empirical modelling	ARDL; DOLS	WDI	Malaysia	Bangladesh	Dynamic impacts of economic growth, renewable energy use, and technological innovation on CO^2^ emissions in Bangladesh
Raihan et al.^20^	Wind power	Econometric/ empirical modelling	ARDL; DOLS	WDI	Malaysia	Bangladesh	Dynamic impacts of economic growth, renewable energy use, urbanisation, industrialisation, technological innovation, and forest area on CO^2^ emissions
Raihan and Tuspekova^21^	Wind power	Econometric/ empirical modelling	ARDL; DOLS	WDI	Malaysia	Peru	Dynamic impacts of economic growth, renewable energy use, and agricultural land expansion on CO^2^ emissions
Horobet et al.^22^	Wind power	Econometric/ empirical modelling	Dynamic system-GMM panel model	OWID, WDI	Romania	163 countries, (not specified)	Contribution of nuclear, fossil (coal, oil, and gas), and renewable (hydro, solar, wind, biofuel) electricity sources to pollution measured as tonnes of CO^2^
Obobisa^23^	Wind power	Econometric/ empirical modelling	AMG; CCEMG	WDI	China	73 countries^c^	Financial development, renewable energy consumption, fossil fuel energy consumption, and economic growth effect on CO^2^ emissions
Cheng and Yao^24^	Wind power	Econometric/ empirical modelling	OLS; MG; CCEMG; AMG; PMG; DFE	China Statistical Yearbook, China Energy Statistical Yearbook, CEIC, PSS-System by CNIPA	China	China (30 provinces^d^)	Impact of renewable energy technology innovation on carbon intensity with provincial panel data in China
Cheng et al.^25^	Wind power	Econometric/ empirical modelling	OLS; fixed- effect panel quantile regression	WDI, OECD	China, UK	BRIICS	The effects of six determinant variables (renewable energy supply, development of environmental patents, economic growth, exports, foreign direct investment and domestic credit to the private sector) on the CO^2^ emissions per capita from 2000 to 2013 for the BRIICS countries.
Sun and Dong^26^	Wind power	Economic modelling	Multi-hierarchy meta-frontier DEA	IEA, BP	China	58 countries^e^	CO^2^ reduction inefficiency and potential in nuclear power and three renewable power industries (wind power, solar power, hydro power) in 58 countries.
Song and Chen^12^	Wind power	Case study/ observational	Descriptive statistics	Chongming statistical yearbooks	China	China, Chongming island	Energy transition analysis of Chongming’s world- class ecological island.
Zhao, L. et al.^13^	Wind power	Dynamic risk transmission modelling	Network modelling; cascading failure framework and linear threshold model	UN COMTRADE	China	211 countries^f^	Mitigation of risk in the global wind turbine trade network.
Handayani et al.^27^	Wind power	Energy system modelling	LEAP model	PLN, DEN, DJK-ESDM	The Netherlands, Indonesia, Australia	Indonesia	Five scenarios for the Java-Bali electricity system’s expansion to satisfy the projected future demand to achieve renewable energy targets.
Govindarajan and Ganesh^28^	Wind power	Modelling	Evaluation framework: building a renewable energy index based on 3 composite indicators	Bespoke online portal	India	India,45 cities (not defined)	Climate Smart Cities Assessment Framework on the use of renewable energy for electricity in 45 Indian cities under smart city mission.
Ifaei et al.^29^	Wind power	TESE analysis; *MCDM approach*	Deep and stacked neural networks; HOMER; WEC nexus model; multi-variate statistical analysis; FA-TOPSIS; stochastic linear mathematical model	KMA, KPX, K-water, KEPCO, KOSIS; peer-reviewed papers	South Korea, Australia	South Korea	Sustainability challenges in the Korean energy sector.
Sun et al.^10^	Wind power; transportation	Econometric/ empirical modelling	CCEMG; AMG; Dumitrescu and Hurlin’s panel causality analysis	WDI; IEA; BP	China, Australia, The Netherlands	5 countries^g^	Role of electric vehicles in alleviating environmental pollution and impact of using renewable energy on CO^2^.
Gilmore et al.^11^	Wind power; hydrogen energy	Modelling	TSSolver	Australian Energy Market Operator	Australia	Australia	Optimal mix of firming technologies (pumped hydro, batteries and ‘zero emission’ open-cycle gas turbines) so that Australia’s National Electricity Market can be supplied by 100% variable renewable energy.
Calvillo and Turner^30^	Transportation	Energy system modelling	Integrated MARKAL-EFOM (TIMES); UKTM	UK DfT, IEA, ENSG, Scottish Power, National Grid, Bloomberg New Energy Finance, peer-revied papers	UK	UK	Impacts of the planned large-scale EV rollout in the UK in terms of network investments, changes in fuel use, fuel cost and emissions;
Logan et al.^31^	Hydrogen energy; transportation	Modelling	TEAM-UK; further scenario modelling developed by the authors	BEIS, CCC, World Nuclear Association, National Grid	UK, Australia	UK	Use of electric and hydrogen buses to reduce greenhouse emissions.
Qadeer et al.^32^	Hydrogen energy	Econometric/ empirical modelling	Quantile-on-quantile regression	CSIRO, daily market index of Australia, Australian Parliament	China; Malaysia; Saudi Arabia	Australia	Hydrogen energy for reducing emissions.

*AMG* Augmented mean group, *ARDL* Autoregressive Distributed Lag, *BEIS* Department for Business, Energy and Industrial Strategy, *BRICS* Brazil, Russia Federation, India, Indonesia, China and South Africa, *CCEMG* Common Correlated Effects Mean Group, *CCC* Committee on Climate Change, *CNIPA* China National Intellectual Property Administration, *CSIRO* Commonwealth Scientific and Industrial Research Organization, *DEA* data envelopment analysis, *DEN* National energy Council of Indonesia, *DFE* Dynamic Fixed Effect, *DfT* Department for Transport, *DJK-ESDM* Ministry of Energy and Mineral Resources of Indonesia, *DK-SE* Driscoll and Kraay estimators, *DARDL* dynamic autoregressive distributed lag simulation, *DOLS* Dynamic Ordinary Least Squares, *ENSG* Electricity Networks Strategy Group, *FA-TOPSIS* factor analysis-based technique for order-preference by similarity to the ideal solution technique, *FMOLS* Fully Modified Ordinary Least Squares, *GMM* generalised method of moments, *HOMER* Hybrid Optimisation Model for Electric Renewables, *IEA* International Energy Agency, *IV-GMM* instrumental variable-generalised method of moments, *KEPCO* Korea electric power corporation, *KMA* Korea meteorological agency, *KOSIS* Korean statistical information service, *KPX* Korea power exchange, *K-water* Korea Water Resources Corporation, *MCDM* multiple-criteria decision making, *MG* Mean Group, *MMQR* method of moments quantile regression, *LEAP* Long-range Energy Alternative Planning, *PMG* Pooled Mean Group, *PLN* Perusahaan Listrik Negara (State Electricity Company of Indonesia), *PSS*-*System* Patent Search and Analysis System, *TEAM-UK* Transport Energy Air Pollution Model for the UK, *TESE* technological, economic, sociological, and environmental, *TSSolver* Time Sequential Solver (linear programming tool), *UKTM* UK TIMES energy system model, *UN COMTRADE* United Nations Commodity Trade Statistics, *WEC* Water-energy-carbon, *WDI* World Development Indicators.

^a^China, the United Kingdom, Italy, Germany, Japan, Indonesia, South Korea, India, France and the United States.

^b^Canada, Mexico, US, Argentina, Brazil, Chile, Colombia, Ecuador, Peru, Trinidad & Tobago, Venezuela, Austria, Belgium, Bulgaria, Croatia, Cyprus, Czech Republic, Denmark, Estonia, Finland, France, Germany, Greece, Hungary, Iceland, Ireland, Italy, Latvia, Lithuania, Luxembourg, Netherlands, North Macedonia, Norway, Poland, Portugal, Romania, Slovakia, Slovenia, Spain, Sweden, Switzerland, Turkey, Ukraine, United Kingdom, Azerbaijan, Belarus, Kazakhstan, Russian Federation, Turkmenistan, Uzbekistan, Iran, Iraq, Israel, Kuwait, Oman, Qatar, Saudi Arabia, United Arab Emirates, Algeria, Egypt, Morocco, South Africa, Australia, Bangladesh, China, India, Indonesia, Japan, Malaysia, New Zealand, Pakistan, Philippines, Singapore, South Korea, Sri Lanka, Thailand, Vietnam.

^c^Algeria, Argentina, Australia, Austria, Bangladesh, Belgium, Benin, Botswana, Brazil, Cambodia, Cameroon, Canada, China, Colombia, Congo, Dem. Rep., Congo, Rep., Cote d’Ivoire, Croatia, Czech Republic, Denmark, Egypt, Eritrea, Ethiopia, Finland, France, Gabon, Germany, Ghana, Greece, Haiti, Hungary, India, Indonesia, Ireland, Italy, Jamaica, Japan, Kenya, Libya, Luxembourg, Malaysia, Mauritius, Mexico, Mongolia, Mozambique, Myanmar, Nepal, Netherlands, Nigeria, Norway, Pakistan, Paraguay, Peru, Philippines, Poland, Portugal, Russian Federation, Senegal, Singapore, South Africa, South Korea, Spain, Sudan, Sweden, Switzerland, Tanzania, Thailand, Togo, Tunisia, United Kingdom, United States, Vietnam, Zimbabwe.

^d^Beijing, Anhui, Chongqing, Fujian, Gansu, Guangdong, Guangxi, Guizhou, Hainan, Hebei, Heilongjiang, Henan, Hubei, Hunan, Inner Mongolia, Jiangsu, Jiangxi, Jilin, Liaoning, Ningxia, Qinghai, Shaanxi, Shandong, Shanghai, Shanxi, Sichuan, Tianjin, Xinjiang, Yunnan, Zhejiang.

^e^Argentina, Australia, Austria, Bangladesh, Belgium, Brazil, Bulgaria, Canada, Chile, China, Chinese Taipei, Colombia, Croatia, Czech Republic, Denmark, Ecuador, Finland, France, Germany, Greece, Hungary, Iceland, India, Indonesia, Iran, Ireland, Israel, Italy, Japan, Kazakhstan, Korea, Lithuania, Luxembourg, Malaysia, Mexico, Netherlands, New Zealand, Norway, Pakistan, Peru, Philippines, Poland, Portugal, Romania, Russian Federation, Slovak Republic, Slovenia, Spain, Sri Lanka, Sweden, Switzerland, Thailand, Turkey, Ukraine, United Kingdom, United States, Venezuela, Vietnam.

^f^Afghanistan, Albania, Algeria, Andorra, Angola, Antigua and Barbuda, Argentina, Armenia, Aruba, Australia, Austria, Azerbaijan, Bahamas, Bahrain, Bangladesh, Barbados, Belarus, Belgium, Belize, Benin, Bermuda, Bhutan, Bolivia (Plurinational State of), Bosnia Herzegovina, Botswana, Brazil, Brunei Darussalam, Bulgaria, Burkina Faso, Burundi, Cabo Verde, Cambodia, Cameroon, Canada, Cayman Islands, Central African Rep., Chad, Chile, China, China, Hong Kong SAR, China, Macao SAR, Christmas Islands, Colombia, Comoros, Congo, Cook Isds, Costa Rica, Côte d’Ivoire, Croatia, Cuba, Curaçao, Cyprus, Czechia, Dem. People’s Rep. of Korea, Dem. Rep. of the Congo, Denmark, Djibouti, Dominica, Dominican Rep., Ecuador, Egypt, El Salvador, Equatorial Guinea, Estonia, Eswatini, Ethiopia, Faeroe Islands, Falkland Islands, Fiji, Finland, France, French Polynesia, Gabon, Gambia, Georgia, Germany, Ghana, Gibraltar, Greece, Greenland, Grenada, Guam, Guatemala, Guinea, Guinea-Bissau, Guyana, Haiti, Honduras, Hungary, Iceland, India, Indonesia, Iran, Iraq, Ireland, Israel, Italy, Jamaica, Japan, Jordan, Kazakhstan, Kenya, Kiribati, Kuwait, Kyrgyzstan, Lao People’s Dem. Rep., Latvia, Lebanon, Lesotho, Liberia, Libya, Lithuania, Luxembourg, Madagascar, Malawi, Malaysia, Maldives, Mali, Malta, Marshall Islands, Mauritania, Mauritius, Mayotte, Mexico, Mongolia, Montenegro, Montserrat, Morocco, Mozambique, Myanmar, Namibia, Nauru, Nepal, Netherlands, New Caledonia, New Zealand, Nicaragua, Niger, Nigeria, North Macedonia, Norway, Oman, Pakistan, Palau, Panama, Papua New Guinea, Paraguay, Peru, Philippines, Poland, Portugal, Qatar, Rep. of Korea, Rep. of Moldova, Romania, Russian Federation, Rwanda, Saint Barthélemy, Saint Helena, Saint Kitts and Nevis, Saint Lucia, Saint Maarten, Saint Pierre and Miquelon, Saint Vincent and the Grenadines, Samoa, San Marino, Sao Tome and Principe, Saudi Arabia, Senegal, Serbia, Seychelles, Sierra Leone, Singapore, Slovakia, Slovenia, Solomon Islands, Somalia, South Africa, Spain, Sri Lanka, State of Palestine, Sudan, Suriname, Sweden, Switzerland, Syria, Tajikistan, Thailand, Timor-Leste, Togo, Tonga, Trinidad and Tobago, Tunisia, Turkey, Turkmenistan, Turks and Caicos Islands, Uganda, Ukraine, United Arab Emirates, United Kingdom, United Rep. of Tanzania, Uruguay, USA, Uzbekistan, Vanuatu, Venezuela, Viet Nam, Wallis and Futuna Islands, Yemen, Zambia, Zimbabwe.

^g^United States of America (USA), China, Germany, France, and Norway.

Fifteen of the studies focused their analysis on one country and the remaining eight on multiple countries (Table 1). The vast majority of the studies (22 out of 23) used a type of modelling for their analysis. Most studies (14 out of 23) employed an econometric methodology in their analysis; three used traditional modelling approaches, and two used energy system modelling. Economic modelling, dynamic risk transmission modelling, and multiple criteria decision-making modelling were used by each of the remaining studies, while there was also one case study. Further details on the methods applied can be found in Table 1.

Only one study^28^ collected primary data for their analysis using a bespoke online portal. The rest of the studies used a mix of sources for their modelling data coming from: publicly available sources, such as the World Development Indicators (WDI) from the World Bank databank^33^ and International Energy Agency (IEA) reports^34^; national authorities/companies, such as the National Energy Council of Indonesia (DEN) and the Korea Electric Power Corporation (KOSIS); data from peer-reviewed papers; and, data from commercial companies such as the PB energy outlook reports^35^ (Table 1).

### Methodological variation and policy recommendation themes

The focus of the interventions and the measured outcomes varied. Two studies^22,28^ did not specify the geographical areas that their analysis focused on. Fourteen studies employed econometric-empirical modelling, and ten^10,14,16,18–23,25^ of them used CO_2_ emissions as their dependent variable, which was the focus of their analysis. Amongst these studies, the independent variables used in the modelling varied substantially (Table 2). Only two independent variables, namely the use of renewable energies and economic growth was used in all studies. Therefore, a direct comparison of the studies’ findings and PRs is not feasible.

**Table 2:** Econometric studies modelling details

Study	Model independent variables	Model dependent variable
Qadeer et al.^32^	GDP (economic growth), trade openness, population	Carbon emissions, methane emissions, nitrous emissions
Zhao et al.^15^	Renewable energy consumption, economic development, industry structure, international trade, and urbanisation	Carbon lock-in
	Industry lock-in, institution lock-in, technology lock-in, and society lock-in	Integrated International Carbon Lock-In Index
Raihan et al.^16^	Economic growth, renewable energy use, urbanisation, industrialisation, tourism, agricultural productivity, and forest area	CO_2_ emissions
Jahanger et al.^14^	Economic growth (GDP), consumption of renewable energy, technology, variable manufacturing sector, energy efficiency	Greenhouse gas emissions (GHGs) measured in Kt of CO_2_ equivalent
Sun et al.^10^	Electric vehicles, GDP, population, urbanisation, renewable energy consumption	CO_2_ emissions
Raihan and Tuspekova^18^	Economic growth, renewable energy use, urbanisation, industrialisation, tourism, agricultural productivity, and forest area	CO_2_ emissions
Raihan et al.^19^	Economic growth, renewable energy use, and technological innovation	CO_2_ emissions
Raihan et al.^20^	Economic growth, renewable energy use, urbanisation, industrialisation, technological innovation, and forest area	CO_2_ emissions
Horobet et al.^22^	Share of electricity generation that comes from fossil fuels (coal, oil and gas combined); nuclear power; solar power, wind power; biofuels; hydropower, GDP. trade openness	Per capita greenhouse-gas emissions produced in the generation of electricity (measured in million tonnes of CO_2_ equivalent); Carbon intensity of electricity production
Raihan and Tuspekova^21^	Economic growth, renewable energy use, and agricultural land expansion	CO_2_ emissions
Obobisa^23^	Financial development, renewable energy consumption, fossil fuel energy consumption, economic growth, and economic growth square.	CO_2_ emissions
Cheng and Yao^24^	Renewable energy technology innovation, industrial structure, energy consumption structure, and urbanisation rate	Carbon intensity (ratio of total CO_2_ emissions to real GRP)
Cheng et al.^25^	Renewable energy supply, development of environment-related technologies, GDP, exports of goods and services, foreign direct investment and domestic credit to private sector	CO_2_ emission per capita
Hossain et al.^17^	GDP, eco-innovation, nuclear energy consumption, fossil fuel consumption and renewable energy consumption	Impact on Load Capacity Factor (Load Capacity Factor (LCF) = Biocapacity per capita/Ecological footprint per capita)

Only two studies^11,30^ defined PRs as primary outcomes. The PRs made by the studies often expanded beyond the scope of this systematic review, only the PRs relevant to climate change mitigation and/or reaching net zero targets were extracted, in line with the eligibility criteria. The full PRs can be found in Supplementary Table 2. In a thematic analysis, four themes of PRs were identified: economic measures, energy measures, public-facing measures and innovation-technology measures. The results of the thematic analysis, along with specific practical examples of PR, are illustrated in Supplementary Figure 1. The majority of the studies presented their PRs in a separate section right after the discussion section, either following or preceding the conclusions section.

### Dual appraisal reveals high risk of bias in evidence and communication

**Fig. 2:**
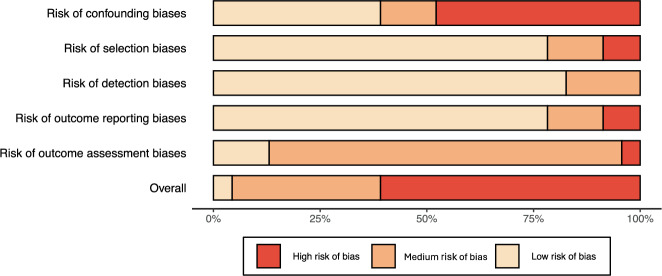
Collaboration for environmental evidence critical appraisal tool (CEECAT) summary of results. Collaboration for Environmental Evidence critical appraisal tool (CEECAT) addresses the risk of bias (RoB) in the five different domains illustrated in the five top bars of the figure. An overall RoB rating is also attributed to each paper. Here, the three ratings of high RoB, medium RoB and low RoB are illustrated by percentage for the entirety of the 23 studies that are included in the systematic review.

**Fig. 3:**
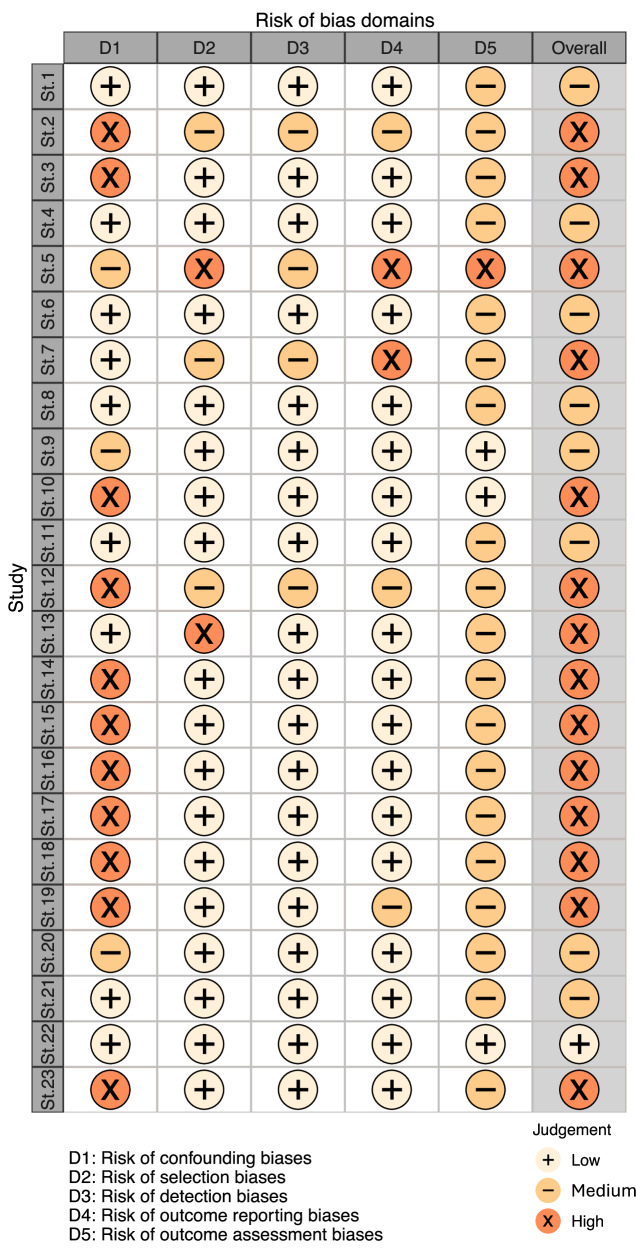
Collaboration for environmental evidence critical appraisal tool (CEECAT) individual study results. The Collaboration for Environmental Evidence critical appraisal tool (CEECAT) addresses the risk of bias (RoB) in five different domains; D1: risk of confounding biases, D2: risk of selection biases, D3: risk of detection biases, D4: risk of outcome reporting biases, and D5: risk of outcome assessment biases. (+) indicates low RoB and (−) indicates medium RoB and (x) indicates high RoB, RoB is examined and presented for each individual study: St.1, Calvillo and Turner^30^; St.2, Cheng and Yao^24^; St.3, Cheng et al.^25^; St.4, Gilmore et al.^11^; St.5, Govindarajan and Ganesh^28^; St.6, Handayani et al.^27^; St.7, Horobet et al.^22^; St.8, Hossain et al.^17^; St.9, Ifaei et al.^29^; St.10, Jahanger et al.^14^; St.11, Logan et al.^31^; St.12, Obobisa^23^; St.13, Qadeer et al.^32^; St.14, Raihan and Tuspekova^21^; St.15, Raihan and Tuspekova^18^; St.16, Raihan et al.^20^; St.17, Raihan et al.^19^; St.18, Raihan et al.^16^; St.19, Song and Chen^12^; St.20, Sun and Dong^26^; St.21, Sun et al.^10^; St.22, Zhao, C. et al.^15^; St.23, Zhao, L. et al.^13^.

Two quality appraisal tools were used in this systematic review, the Collaboration for Environmental Evidence Critical Appraisal Tool (CEECAT)^36^ and the Evidence Communication Rules for Policy (ECR-P) critical appraisal tool^37^, each focusing on different aspects of the studies. The results of the CEECAT assessment are illustrated in Fig. 2. Out of the seven domains (criteria) of the tool, only five were used, as criteria three and four are designed for study types that were not identified nor included in our systematic review (observational and experimental). The highest bias was identified in criterion 1 of the tool, concerning risk arising from confounding biases. Fourteen studies^12–14,16,18–25,28,32^ were found to be of overall high RoB, eight^10,11,17,26,27,29–31^ exhibiting medium RoB, and only one study^15^ was assessed to be of low RoB. The results for each individual study for each criterion are illustrated in Fig. 3.

The domain of confounding biases was identified with the highest potential for risk across the studies (11 out of 23). Nine out of these 11 studies used econometric methods as described in the previous section. The omission or addition of variables in the models that seemingly had very similar research objectives raises significant questions on the existence of confounding parameters and their potential effects on the modelling results. The inconsistent configuration of the models (Table 2) and the lack of supporting justification are reflected in the CEECAT assessment results.

**Fig. 4:**
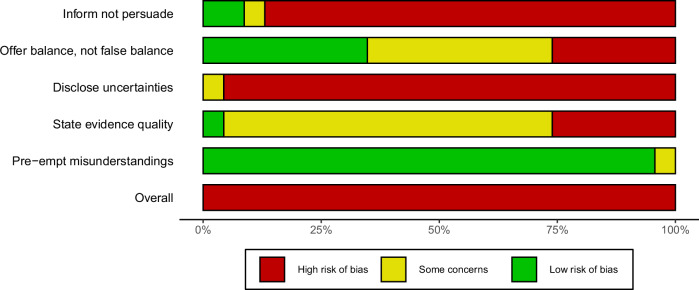
Evidence communication rules for policy (ECR-P) critical appraisal tool summary of results. The Evidence Communication Rules for Policy (ECR-P) critical appraisal tool addresses the risk of bias (RoB) in the five different domains illustrated in the five top bars of the figure. An overall RoB rating is also attributed to each paper. Here, the three ratings of high RoB, some concerns and low RoB are illustrated by percentage for the entirety of the 23 studies that are included in the systematic review.

**Fig. 5:**
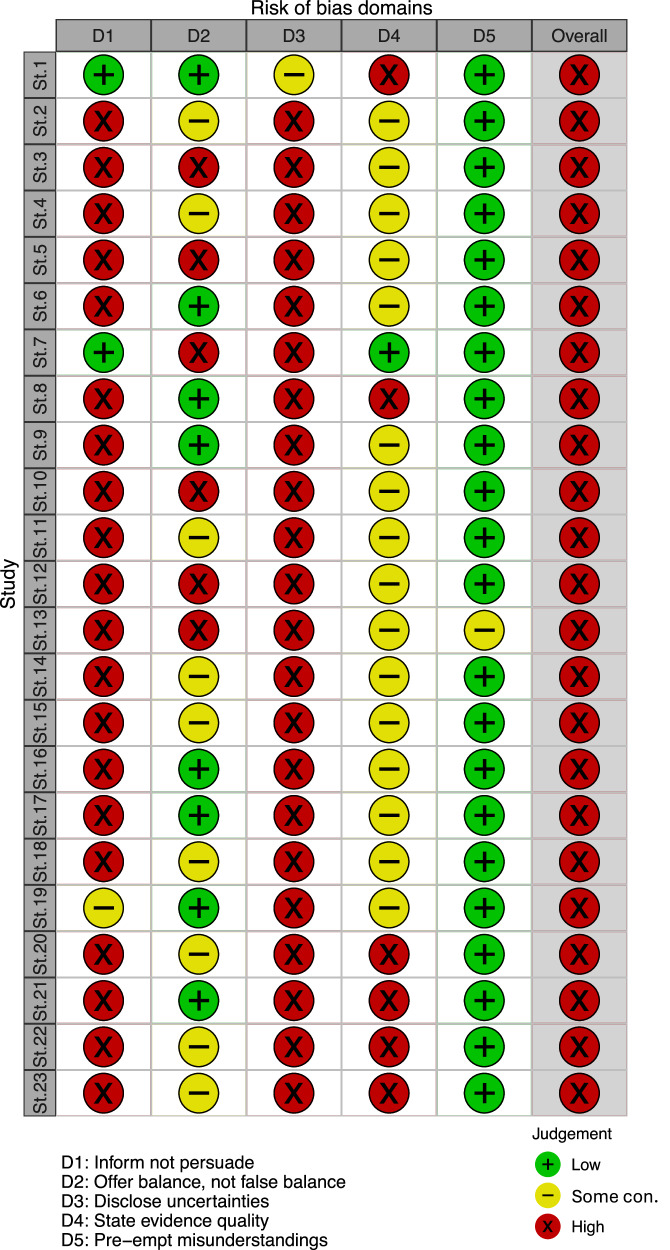
Evidence communication rules for policy (ECR-P) critical appraisal tool, individual study results. The Evidence Communication Rules for Policy (ECR-P) critical appraisal tool addresses the risk of bias (RoB) in five different domains; D1: Inform not persuade, D2: Offer balance, not false balance, D3: Disclose uncertainties, D4: State evidence quality, D5: pre-empt misunderstandings. (x) indicates high RoB, (+) indicates low RoB and (−) indicates some concerns. RoB is examined and presented for each individual study: St.1, Calvillo and Turner^30^; St.2, Cheng and Yao^24^; St.3, Cheng et al.^25^; St.4, Gilmore et al.^11^; St.5, Govindarajan and Ganesh^28^; St.6, Handayani et al.^27^; St.7, Horobet et al.^22^; St.8, Hossain et al.^17^; St.9, Ifaei et al.^29^; St.10, Jahanger et al.^14^; St.11, Logan et al.^31^; St.12, Obobisa^23^; St.13, Qadeer et al.^32^; St.14, Raihan and Tuspekova^21^; St.15, Raihan and Tuspekova^18^; St.16, Raihan et al.^20^; St.17, Raihan et al.^19^; St.18, Raihan et al.^16^; St.19, Song and Chen^12^; St.20, Sun and Dong^26^; St.21, Sun et al.^10^; St.22, Zhao, C. et al.^15^; St.23, Zhao, L. et al.^13^.

Five domains were examined within the ECR-P critical appraisal tool. ECR-P was specifically designed for assessing the communication and the quality of scientific-based PRs as well as their evidence base. This systematic review is the first in which this tool has been used, following extensive piloting and validation. The summary of the assessment is illustrated in Fig. 4, while the assessment for individual studies is presented in Fig. 5.

ECR-P assessment is executed for each domain at two different levels: the study level and the PRs level. Therefore, in the first instance, results are presented separately for each of these two levels. The results of each level are then combined for each domain, and an overall assessment is provided across all domains. In order to achieve the highest transparency and to draw conclusions around the drivers of quality, the assessment results for each lever per domain are also provided. Supplementary Figs. 2 and 3 present the summary assessment for study level and PRs level, respectively, while Supplementary Figs. 4 and 5 present the individual study assessment results. The results for each domain of the ECR-P critical appraisal tool are presented in the following sections.

### Advocacy and the use of emotive language

In the domain *inform, not persuade*, 87% of the studies (20 out of 24) were rated as high risk of bias. This is the second-worst-rated domain in the tool. It should be noted that only 11 out of the 20 studies were assessed for high risk in both study level and PRs level (Supplementary Figs. 4 and 5). At the study level, the areas that drove the high-risk results were concerned with the studies not reporting the limitations of their analysis and lacking a clear connection between their findings and conclusions.

Similarly, in the PRs level, these two areas were also identified as problematic since studies did not report the limitations of their PRs, and did not clearly connect their PRs to the findings of their research. From the studies that did report PRs’ limitations, the majority (10 out of 11) related the reported study limitations to both findings and PRs. Only one study^31^ addressed specifically the PRs’ limitations and recognised that they are contingent on factors that were not part of their analysis.

Almost half of the studies (48%) provided some PRs relevant to their findings but also put forward PRs that were not scientifically based (based on the scientific findings of their study). For example, it was observed that a lot of studies had PRs on promoting environmental consciousness as a way of increasing the use of green energies that would lead to reducing carbon emissions^15,16,18–21^. Although, these PRs might ‘make sense’ in the context of tackling climate change, they were not connected to the studies’ scientific analysis and findings.

In the PRs level, a third area was identified as problematic. Six studies were found to have used emotive language in communicating their PRs, indicating advocacy rather than neutral scientific reporting. Examples of emotive language included: “*Climate-minded policymakers should implement unprecedented reforms and wean their citizens off fossil fuels…*”^23^, “ *…the use of obsolete, polluting technologies must be forbidden*.”^16^, “*Alternatives that are more ecologically friendly should be used in place of obsolete and incompetent technology*”^32^.

On the other hand, the studies overall did very well in clearly reporting their aims and objectives for both research outcomes and PRs, proposing ways to tackle the reported study limitations in future research, avoiding the use of emotive language in their findings and conclusions, and using accessible language in their PRs.

### Balance and the consideration of policy complexity

In the domain *offer balance, but not false balance*, 35% and 39% of the studies were rated as low RoB and some concerns, respectively (Fig. 5). On the study level, only two studies^22,28^ were rated as high RoB. Both these studies included a lot of different geographical areas (163 countries and 45 cities), but didn’t specify them or report how and why they were chosen for their analysis. For this domain and level, the tool asked whether a reporting guideline had been used. To the best of our knowledge, a discipline-specific reporting guideline does not exist for this field, therefore, the use of a guideline was not assessed.

The PRs level in this domain focuses on two areas: whether the authors acknowledge the inherent complexity of PRs and thus consider their potential multiple implications, and whether they have an overview of the current policies that are in place (or a lack thereof) for the issues they are researching. Indeed, most studies offered multiple PRs and in different policy areas, but only one study^32^ considered the potential negative implications of their PRs. Nineteen studies exhibited knowledge of current policies, but only nine of them considered the implications of not changing the *status quo* (Supplementary Fig. 5). High risk of bias was attributed to the four studies that did not mention the current policy status.

### Disclosing and addressing uncertainty

The domain *disclose uncertainties* was rated with the highest risk of bias across all studies. Indeed, 96% were rated as of high RoB and 4% as exhibiting some concerns (Fig. 5). The study level focuses on reporting uncertainties in study findings and proposing ways to reduce them in the future. Most studies (20 of 23) did indeed report uncertainties, but only one^30^ suggested a way to alleviate them.

In the PRs level, studies overwhelmingly did not address uncertainties. Only one study discussed uncertainties, but only briefly and partially, stating ‘*Therefore, we see this analysis as necessary first step for further research on the full implications of the EV rollout in the energy system and the wider economy*’^30^. It should be noted that the study did not propose a way forward in addressing uncertainties in the future in any specific way.

An important distinction has to be made between the concepts of limitations and uncertainties. Limitations are found in methodology, data input and data analysis. Ultimately, these affect and ‘limit’ the study’s outputs. Uncertainties largely relate to the difference between the research findings and the ‘true values’ and help readers understand the degree of confidence in the study findings.

### Evidence quality and the lack of reflection

Most of the studies (70%) raised only some concerns about bias in the domain *state evidence quality*. There is a clear mismatch between the rating of the study level and the PRs level (Supplementary Figs. 2–5). In the study level, 17 studies appear to have used high-quality data inputs, but only a small subsection of them actively discussed their quality. Only three studies went into detail about how data collection processes ensured data quality^28^, how data production was relevant for their analysis^31^, or acknowledged poor quality for a subset of their data^11^. On the other hand, six studies^10,13,15,17,26,30^ reported that they had used data of questionable quality, mainly coming from commercial companies’ reports (see Table 1), with no consideration given to their quality or to the obvious potential for competing interests. This led to these six studies having a high RoB rating at the study level. No specific metrics for data quality were used by any studies.

On the PRs level, the focus was on whether the studies considered the quality of the study findings that formulated the evidence base for the PRs. Only one study actively discussed this issue, stating: ‘*Our study, like any other research, has limitations. They are caused by the data used and the availability of data, the time period under consideration, and the variables included in the models. All of these constraints can be addressed in future research, as well as the impact of specific policies on electricity-generated pollution*’^22^. Not considering evidence quality for PRs led to nineteen studies being rated as of high RoB in the PRs level of this domain.

### Misunderstandings and clarity of communication

The last domain examined was *pre-empt misunderstandings*. This domain focused on preventing misunderstandings, to help inoculate against misinformation those who were using the evidence. This was the domain that was rated best across all studies for both study level and PRs level. Only one study^32^ was found to raise some bias concerns, while the remaining 22 were rated as of low RoB. On the study level, the quality of the communication was assessed as to whether studies tried to pre-emptively address potential misunderstandings about the study findings and conclusions and ultimately, if any ambiguity was identified. In the PRs level, in addition to assessing the clarity of the PRs themselves, the targeted policy makers also needed to be defined. Both areas were rated as low RoB across all studies.

### ECR-P overall quality reveals the prevalence of a high risk of bias

**Fig. 6:**
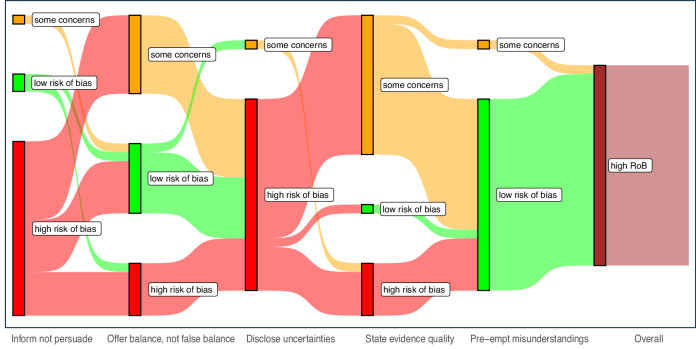
Evidence communication rules for policy (ECR-P) critical appraisal tool, individual study rating flow. The Evidence Communication Rules for Policy (ECR-P) critical appraisal tool addresses the risk of bias (RoB) in the five different domains that are illustrated at the bottom of the figure, followed by an overall RoB rating. Rating is either high RoB, some concerns or low RoB.

All studies were rated as having an overall high RoB within the ECR-P critical appraisal tool. Studies did better in some domains than others, but in the synthesis of the domains, all studies were rated with high RoB in at least one of them, thus resulting in an overall high RoB rating. These results indicate poor quality of PRs’ formulation and communication. The flow of the ratings through the five domains is illustrated in Fig. 6. The two worst-rated domains were domain 1: inform not persuade, and domain 3: disclose uncertainties.

The critical appraisal process revealed that there were specific questions of the ECR-P tool that all studies felt short in addressing. The question that had the most negative responses (either “no” or “probably no”) was 3.2 ‘*Were uncertainties of the policy recommendations reported?*’, with 22 negative responses; followed by the conditional question 3.1.1 (If Y/PY to 3.1) ‘*Did the study propose ways to reduce uncertainties in the future?*’, with 19 negative responses; and question 4.2 ‘*Was the quality of the study findings, that formulated the evidence base for the policy recommendations, considered?*’, also with 19 negative responses.

The domain that was rated best across all studies was domain 5: Pre-empt Misunderstandings. The three studies that were best rated were by Calvillo and Turner^30^, Horobet et al.^22^, and Song and Chen^12^. The study that was rated best across all levels and domains was one by Calvillo and Turner^30^, rated with low RoB in three domains (1, 2 and 5), one domain with some concerns (3) and one with high RoB (4). This study focused on the rollout of electric vehicles in the UK. Interestingly, it was one of the two studies which had PRs as their main research outcome (the other being the study by Gilmore et al.^11^), and it was the only study that addressed uncertainties in their PRs. Calvillo and Turner^30^ were only rated poorly in domain 4. Regarding the study level, although they used secondary evidence as inputs for their energy system modelling from commercial companies (including Scottish Power and Bloomberg New Energy Finance), they did not consider the quality of their data sources. The issue was propagated in the PRs section. On the other hand, Song and Chen^12^ were also rated poorly in only one domain, but here it was domain 3, as the study did not address uncertainties in their findings or their PRs. This was the only case study included in the systematic review. Horobet et al.^22^ was the only other study (apart from Calvillo and Turner^30^) that was rated with low RoB in three separate domains (1, 4 and 5). Calvillo and Turner^30^ and Horobet et al.^22^ were the only two studies that were rated low RoB in the PRs level in domain 1, providing high-quality communication across the areas addressed in this domain (Supplementary Table 3).

There was sufficient agreement between the overall ratings of the two critical appraisal tools. The ratings were the same for 14 out of the 23 studies and for two additional studies when comparing the overall rating from CEECAT and the study-level rating from ECR-P. As expected, the agreement was much stronger for the studies that were rated poorly in the CEECAT. The direction of the rating was different (low versus high risk of bias) for only one paper. Agreement between the tools is presented in Supplementary Table 4.

## Discussion

To our knowledge, this is the first systematic review to focus on the communication and quality of scientific-based PRs in any scientific field. The domains that exhibited the greatest weaknesses were maintaining a non‑advocacy stance and disclosing uncertainties, especially when it came to uncertainties around the PRs. The review also identified a distinct superiority of communication and quality of study findings and conclusions over the communication and quality of PRs. Overall, the current status of PRs was found to be of poor quality.

The importance of these findings is highlighted by recent research demonstrating that timely access to high-quality scientific evidence is a key determinant of evidence uptake in policymaking^18^. Furthermore, the study^30^ in the review with the highest-quality PRs predefined them as a primary research objective, reinforcing the conclusion that most studies approached PRs as an afterthought. These findings emphasise the need to strengthen the quality of communication in scientific evidence-based policymaking across the global climate governance landscape.

One of the most important findings of this systematic review is the complete lack of studies in engineering or experimental environmental science that put forward PRs for tackling climate change, within the scope of the areas included in our review questions, i.e. wind power, hydrogen energy and transportation. It is surprising that no such research exists, considering how topical the issues around climate change are and the sharp increase in peer-reviewed paper publications in every scientific field. Our exhaustive searches in four different databases did not identify any such studies. This finding suggests that including PRs in these fields is still far from being the norm. Overall, 23 studies were included in this methodological systematic review. The number of included papers provides an important insight into the research gap as well as the state of research evidence at this specific point in time in this very topical research field.

Studies often started with very specific and, in many cases, practical PRs (e.g. fiscal measures) but ended up rounding up the PRs section with a sort of ‘wish list’ of recommendations that would be considered common ground in the climate change realm but were not connected to their analysis. It is not clear if the authors felt compelled to include these PRs so that they conform to the consensus view or if they were simply stating their opinion. Nevertheless, putting forward non-scientific-based PRs reduced the overall trustworthiness of the studies.

The review also identified a shortcoming in addressing the limitations and the uncertainties of studies and their PRs. In addition, evidence quality was not actively discussed by the majority of the studies neither in the study findings nor in the PRs. Standardised metrics of data quality were not used by any of the studies. Transparency regarding limitations, uncertainties and evidence quality speaks to the trustworthiness of the study^2^. Transparent reporting, which is championed by ECR-P, might be maliciously used by others to cast doubt^38^. Nevertheless, this should not deter researchers from reporting them as lack of transparency can lead to long term issues of trust.

Although, it could be expected that emotive language would be avoided in scientific literature, surprisingly, it was identified in more than a quarter of the studies (26%). The use of emotive language in PRs could be ever more alarming as researchers might be tempted to use ‘stronger’ language to persuade potential policymakers.

Only one study^32^ considered the potential negative implications of their PRs, thus offering a balanced communication. Policies are inherently multifactorial and can have multiple effects. A consideration of multiple outcomes of the policy recommendation shows that researchers acknowledge the complexity of policies. In many policy areas, it might be the case that a policy is already in place for the issue the study is focusing on. Knowledge of the current policy, or, indeed, the absence of one, is essential for putting forward future PRs^39^.

The majority of studies clearly presented their PRs in a separate section. Indeed, research papers that include a summary with clear recommendations are more likely to be used as evidence in policy making^40^. Pre-empting misunderstandings was rated best for all studies. This was not because studies actively tried to address issues, but because they used practical examples, thus minimising clarity problems that might arise. Clear and unambiguous reporting of study findings, conclusions and PRs is essential and is often a topic of critique within the peer-review process. Anticipating and pre-emptively inoculating against misunderstandings, misinformation, or even disinformation is key. Research targeting the needs of decision makers facilitates its use in policymaking^3^. The first step in pre-empting misunderstandings in PRs is to correctly identify the audience, their needs and expectations.

Five^16,18–21^ of the included studies in the systematic review shared at least the lead author. The inclusion of studies by the same authors might have skewed our results and is recognised as a limitation of this systematic review.

Formulating and providing scientific-based PRs as part of peer-reviewed papers can help tackle knowledge exchange barriers between scientists and policymakers. These PRs can truly serve evidence-based policymaking only if they are based on standards of the highest quality in conducting and communicating research. According to the ratings of the ECR-P critical appraisal tool, the PRs of all studies were found to be of low quality. There was also a clear trend that studies were rated much better at the study level than at the PRs level. This observation might be attributed to the fact that most authors are more accustomed to reporting study findings and conclusions than PRs. A lack of know-how in formulating and communicating PRs might explain this disparity.

In addition, there are no reporting guidelines for this scientific field nor for scientific-based PRs - in many disciplines their use is the norm. These guidelines denote the structure and the minimum information that a paper should report to allow readers to make independent judgements and draw conclusions. Increasingly, scientific journals require a documented use of such reporting guidelines as a prerequisite for accepting a manuscript for peer-review (e.g. PRISMA guideline for reporting systematic reviews^41^, CONSORT guidelines for reporting parallel group randomised trials^42^, CHEERS guidance for Health Economic Evaluation^43^, COREQ criteria for reporting qualitative research^44^, etc.).

There is a clear need for reporting guidelines in scientific-based PRs. The findings of this systematic review provide insights into the current status of PRs and identify areas where authors should be more attentive when communicating their PRs. These findings, in conjunction with the ECR-P critical appraisal tool, can be used as a foundation for a reporting guideline that will promote research integrity.

Authors should use the same scientific rigour in the development and communication of their PRs as in any other section of their studies, meticulously building the trustworthiness of their scientific outputs. Improving the quality of reporting and communication has been shown to affect the impact in policymaking and decision-making in general^45^. The findings of this systematic review focus on exemplars of key policymaking areas within the green agenda. Further research in other scientific fields is needed in order to deepen our understanding of the communication and quality of PRs and to have a more holistic appreciation of current challenges and ways forward.

## Methods

Systematic reviews are executed following a protocol which sets out the scope and the methods to be used^46,47^. The protocol is prepared and made publicly available before conducting the systematic review^48^. As such, they adhere to explicit, pre-defined and reproducible methods. A key difference between systematic reviews and ‘traditional’ literature reviews is that the former aim to answer specific research question/s rather than provide an overview of the literature. These questions are pre-defined in the protocol. In the present work, the research questions were formulated using FINER criteria as guidance. These criteria state that questions should be Feasible, Interesting, Novel, Ethical and Relevant^49^.

This systematic review followed the guidance specified in the Cochrane Handbook for Systematic Reviews of Interventions^47^ and guidance from the Centre for Reviews Dissemination^46^. Reporting was based on the PRISMA (Preferred Reporting Items for Systematic Reviews and Meta-Analyses) statement^41^ as evidenced in the PRISMA checklist reported in Supplementary Table 5. The research protocol was published on the Research Registry (UIN: reviewregistry1795).

### Eligibility criteria and search strategy

The scope of a systematic review is defined by the review questions, which can be framed in terms of specific characteristics of the studies that are eligible to be included in the review. These characteristics are often described using the PICOS acronym (Population, Intervention, Comparator, Outcome/s, Study type)^46^ and are set out during the development of the protocol (see UIN: reviewregistry1795).

According to the eligibility criteria, only research with an environmental focus in the areas of green energy, specifically wind power and hydrogen energy, and transportation (road vehicles) was included. The scope was further tailored to the needs of a wider project focusing on offering solutions for intractable engineering problems for net zero using AI and machine learning tools, in the areas of wind farms, safe hydrogen operations and road transport^50^. These are significant policy areas where there are multiple policy-based questions being asked.

Research design could either be experimental, observational, computational or relate to case studies, including those based on machine learning and artificial intelligence. In addition, only studies reporting on an outcome of policy implications/recommendations for climate change mitigation or/and reaching net zero targets (decarbonisation) were to be included. There was no limitation on the study publication language but only studies published in the last five years were included. The five-year limit is in line with the scope and the research objectives of this systematic review, focusing on the current state of evidence and PR communication. The time limit was also reflected in the search strategy. Systematic reviews were only included for reference checking and background information. Reviews, opinion pieces, commentaries, perspective articles, economic studies and any type of conference abstracts were excluded.

Multiple online databases/sources were searched from 2019 to the present. The final searches were executed on the 8th of February 2024. The Web of Science core collection and Scopus were searched as multidisciplinary sources. In addition, subject-specific databases, GeoRef and GreenFile, were also searched to identify any additional geoscience and ecology literature. Separate search strategies were developed for each database according to its configuration. In order to validate that all relevant studies were included, the reference lists of the systematic reviews that were identified by the search strategy were hand-searched. Search strategies were piloted before the final version to achieve a balance between sensitivity and specificity. There were no restrictions on publication status or publication language. Search terms included: net zero; climate change; renewable/sustainable/green; energy/power/fuel; transportation; wind; hydrogen; policy recommendation/implication/suggestion; policymaking; policy makers, etc. The full search strategy, including the applied Boolean operators, is reported in Supplementary Table 6.

### Selection and data collection process

The citations of articles identified by the search strategy were extracted and managed using EndNote software. EndNote was also used for the initial de-duplication of the records. Screening of the identified records was executed independently by two reviewers (E.D. and A.S.) in two stages. In the first stage, titles and abstracts were screened against the eligibility criteria. Discrepancies between the reviewers were resolved by consensus and by seeking advice from a third reviewer (J.A.). Full papers were consequently retrieved for the records which fulfilled the eligibility criteria. These full papers were then screened in the second screening stage following the same method.

Data were extracted in pre-piloted data extraction forms (Excel) by one reviewer (E.D.) and checked by a second reviewer (A.S.). Disagreements between the reviewers were resolved by consensus and by seeking advice from a third reviewer (J.A.). The following data items were extracted: study design; discipline; language; methodology; methods; data source; area of research; country; study aims; funding; primary/secondary outcomes; study conclusions; policy recommendations; limitations. The key outcome the systematic review focused on was PRs.

### Study risk of bias assessment

Every systematic review must include a quality appraisal of the included papers’ methodology^47,48^. This procedure of examining the study’s internal validity is also often termed risk of bias (RoB) assessment. Bias in this context refers to the introduction of systematic error (or deviation from the truth) in the results of a study^47^. Quality appraisal is executed using standardised tools, which are essentially checklists setting out criteria that should be met to demonstrate methodological quality^48^. There is a variety of tools available which are tailored to the type, design and context of the study. The results of the quality appraisal guide the evidence synthesis.

Two quality appraisal tools were used in this systematic review. RoB assessment of the individual studies’ findings was based on CEECAT Version 0.3^36^. The scope of this tool is environmental management research, including sustainable energy and consumption, as well as broader contexts of environmental sustainability^36^. Five criteria of CEECAT were used: risk of confounding biases, risk of selection biases, risk of detection biases, risk of outcome reporting biases, and risk of outcome assessment biases. Each criterion is examined by using a series of standardised questions in the form of a checklist. Supporting information is provided for each response. Quality ratings are attributed by CEECAT for each of the five criteria, followed by an overall judgement about RoB for the study findings^36^. The ratings are low RoB, medium RoB and high RoB, denoting high quality, medium quality and low quality, respectively. The rating of a study is overall low RoB when all the criteria were considered low RoB, the rating is overall medium RoB when at least one criterion was considered medium RoB and overall high RoB when at least one criterion was considered high RoB.

In line with the methodological focus of this systematic review, the ECR-P critical appraisal tool^37^ was also used. This is a novel critical appraisal tool for assessing the quality of the policy recommendations and the quality of their evidence base, and how well these have both been communicated. The conceptualisation of the tool was based on the five rules for evidence communication as developed by the Winton Centre for Risk and Evidence Communication^2^. The rules are: inform, not persuade; offer balance, not false balance; disclose uncertainties; state evidence quality; inoculate against misinformation. The development of the tool was carried out by an interdisciplinary team and followed a robust and transparent methodology^47,48,51^. ECR-P was piloted in this systematic review. As such, the formulation of the tool was based on the empirical evidence examined in this systematic review, in addition to theoretical reflections and previous experience in policymaking environments. Full details of ECR-P are presented in a methodology paper^37^.

The structure of ECR-P is domain-based, with each domain mapping to one of the five rules for evidence communication. The tool is a checklist consisting of 25 signalling questions designed to obtain essential information for the critical appraisal process. The available responses are ‘Yes’ and ‘Probably Yes’ (associated with high-quality outcomes) and ‘No’ and ‘Probably No’ (associated with low-quality outcomes). A ‘No information’ response is used when not enough information is available to reach a judgement; ‘not applicable’ is used when a signalling question is connected to a previous one that has not been answered positively. Responses are justified by providing supporting information from the paper under examination. The judgement for each domain is based on the responses to the signalling questions. The judgement options are: low RoB; Some concerns and high RoB, corresponding to high, moderate and low quality. An overall quality judgement is consequently derived by examining the individual domain-based judgements. The worst rating across the five domains is carried over to the overall RoB judgement. Therefore, a study can only be overall Low RoB when all domains are rated Low RoB. The critical appraisal tool can also be found in Supplementary Table 3.

Quality assessment, using both tools, was executed by one reviewer (E.D.) and checked by a second (A.S.). Any discrepancies were resolved by consensus. A third reviewer (J.A.) was consulted when issues remained after consensus. The results of the quality appraisal are used in evidence synthesis, addressing individual study quality as well as the quality of the overall body of evidence.

The purpose of using two critical appraisal tools in this systematic review is twofold. First, the two tools inherently focus on different elements of the papers, thus complementing each other and ensuring that all aspects of validity and communication are assessed. Second, ECR-P is a new tool piloted in this paper; the use of CEECAT, which is a tool that has been used and validated in many reviews, strengthens the methodological rigour of the review and provides an additional safeguard. Agreement between the two tools was explored tabularly and narratively.

### Evidence synthesis

The extracted data were only suitable for narrative synthesis and analysis. Narrative synthesis here refers to the formal process that is based on the four elements of the framework proposed by the Centre for Reviews and Dissemination^46^. The first element is developing a theoretical context addressing how, why and for whom the intervention under examination works. Here, the intervention is the policy recommendations. The second element is developing a preliminary synthesis of findings, which includes bringing the extracted data together, organising and describing them systematically. The third element is exploring relationships within and between studies. In this stage, there is an attempt to identify patterns and to explore relationships between characteristics of individual studies and their findings and between different studies. The fourth element is assessing the robustness of the synthesis. Here, the overall strength of the evidence is examined, looking at the methodological quality of the included studies and the credibility of the narrative synthesis outcome^52^.

The extracted data are presented in tabular form and figures and summarised in text. R (version 3.6.0)^53^ was used for visualisation of results via RStudio (RStudio version 2024.4.2 + 764)^54^ using the additional packages dmetar^55^, robvis^56^, ggsankey and ggplot2^57^.

## Supplementary information

Supplementary information

## Data Availability

All data generated or analysed during this study are included in this published article. The policy recommendations reviewed in this study are reported in Supplementary Table 2. All the papers analysed in this systematic review are publicly available, peer-reviewed papers.
