# Supplementary material for: Blurring evidence with advocacy: a systematic review of policy recommendations for net zero
Source: NPJ Environ Soc Sci. 2026 Jun 9;1(1):6. doi: 10.1038/s44432-026-00012-6 (PMC13277626; doi:10.1038/s44432-026-00012-6)
Supplement: Supplementary file 1 — Supplementary information [file 44432_2026_12_MOESM1_ESM.pdf]

Supplementary information

Communication of evidence and policy recommendations: A systematic review of energy and transport research for net zero

Evangelos Danopoulos[1], Aarushi Shah[1], Claudia R. Schneider[1,2], John A. D. Aston[1]

[1]Statistical Laboratory, Department of Pure Mathematics and Mathematical Statistics, University of Cambridge, Cambridge, United Kingdom

[2]School of Psychology, Speech and Hearing, University of Canterbury, Christchurch, New Zealand

Corresponding author:

Evangelos Danopoulos, [1]Statistical Laboratory, Department of Pure Mathematics and Mathematical Statistics, University of Cambridge, United Kingdom

Email: en406@cam.ac.uk

Supplementary Table 1. Reasons for excluding studies at the full paper screening stage.

Intervention
Adebayo, T. S., Ullah, S., Kartal, M. T., Ali, K., Pata, U. K., & Ağa, M. (2023). Endorsing sustainable development in BRICS: The role of technological innovation, renewable energy consumption, and natural resources in limiting carbon emission. Science of the Total Environment, 859. doi:10.1016/j.scitotenv.2022.160181
Amin, N., Shabbir, M. S., Song, H. M., & Abbass, K. (2022). Renewable energy consumption and its impact on environmental quality: A pathway for achieving sustainable development goals in ASEAN countries. Energy & Environment, 19. doi:10.1177/0958305x221134113
Azam, A., Rafiq, M., Shafique, M., & Yuan, J. (2022). Towards Achieving Environmental Sustainability: The Role of Nuclear Energy, Renewable Energy, and

ICT in the Top-Five Carbon Emitting Countries. Frontiers in Energy Research, 9. doi:10.3389/fenrg.2021.804706

Bigerna, S., Bollino, C. A., & Polinori, P. (2021). Convergence in renewable energy sources diffusion worldwide. Journal of Environmental Management, 292. doi:10.1016/j.jenvman.2021.112784

Bozkaya, Ş., Duran, M. S., & Awan, A. (2023). Technological innovations in environmental sustainability: A quantitative exploration of their impact on carbon dioxide emissions. Natural Resources Forum. doi:10.1111/1477-8947.12354

Chen, H., Lu, J., & Obobisa, E. S. (2023). Striving towards 2050 net zero CO2 emissions: How critical are clean energy and financial sectors? Heliyon, 9(12). doi:10.1016/j.heliyon.2023.e22705

Das, A., Saini, V., Parikh, K., Parikh, J., Ghosh, P., & Tot, M. (2023). Pathways to net zero emissions for the Indian power sector. Energy Strategy Reviews, 45. doi:10.1016/j.esr.2022.101042

Edziah, B. K., Sun, H., Adom, P. K., Wang, F., & Agyemang, A. O. (2022). The role of exogenous technological factors and renewable energy in carbon dioxide emission reduction in Sub-Saharan Africa. Renewable Energy, 196, 1418-1428. doi:10.1016/j.renene.2022.06.130

Elmanakhly, F., DaCosta, A., Berry, B., Stasko, R., Fowler, M., & Wu, X. Y. (2021). Hydrogen economy transition plan: A case study on Ontario. AIMS Energy, 9(4), 775-811. doi:10.3934/ENERGY.2021036

Gota, S., Huizenga, C., Peet, K., Medimorec, N., & Bakker, S. (2019). Decarbonising transport to achieve Paris Agreement targets. Energy Efficiency, 12(2), 363-386. doi:10.1007/s12053-018-9671-3

Ibrahim, R. L., Awosusi, A. A., Ajide, K. B., & Ozdeser, H. (2023). Exploring the renewable energy-environmental sustainability pathways: what do the interplay of technological innovation, structural change, and urbanization portends for BRICS? Environment, Development and Sustainability. doi:10.1007/s10668-023-03917-3

Karmaker, S. C., Chapman, A., Sen, K. K., Hosan, S., & Saha, B. B. (2023). Renewable Energy Pathways toward Accelerating Hydrogen Fuel Production: Evidence from Global Hydrogen Modeling. Sustainability (Switzerland), 15(1). doi:10.3390/su15010588

Lawal, A. I. (2023). Determinants of Renewable Energy Consumption in Africa: Evidence from System GMM. Energies, 16(5). doi:10.3390/en16052136

Luh, S., Ramachandran, K., McKenna, R., Schmidt, T. J., & Kober, T. (2023). How, where, and when to charge electric vehicles – net-zero energy system implications and policy recommendations. Environmental Research Communications, 5(9), 095004. doi:https://doi.org/10.1088/2515-7620/acf363

Mirziyoyeva, Z., & Salahodjaev, R. (2023). Renewable energy, GDP and CO2 emissions in high-globalized countries. Frontiers in Energy Research, 11. doi:10.3389/fenrg.2023.1123269

Padhan, H., Ghosh, S., & Hammoudeh, S. (2023). Renewable energy, forest cover, export diversification, and ecological footprint: a machine learning application in moderating eco-innovations on agriculture in the BRICS-T economies. Environmental Science and Pollution Research. doi:10.1007/s11356-023-27973-4

Phoumin, H., Kimura, F., & Arima, J. (2021). Asean's energy transition towards cleaner energy system: Energy modelling scenarios and policy implications. Sustainability (Switzerland), 13(5), 1-29. doi:10.3390/su13052819

Pollitt, M. G., & Chyong, C. K. (2021). Modelling net zero and sector coupling: Lessons for european policy makers. Economics of Energy and Environmental Policy, 10(2), 25-40. doi:10.5547/2160-5890.10.2.MPOL
Raihan, A., & Tuspekova, A. (2022). Towards sustainability: Dynamic nexus between carbon emission and its determining factors in Mexico. Energy Nexus, 8. doi:10.1016/j.nexus.2022.100148
Raymand, F., Ahmadi, P., & Mashayekhi, S. (2021). Evaluating a light duty vehicle fleet against climate change mitigation targets under different scenarios up to 2050 on a national level. Energy Policy, 149, 10. doi:10.1016/j.enpol.2020.111942
Santos, G., & Smith, O. (2023). Electric vehicles and the energy generation mix in the UK: 2020–2050. Energy Reports, 9, 5612-5627. doi:10.1016/j.egyr.2023.03.114
Shen, Y., Li, X., & Hasnaoui, A. (2021). BRICS carbon neutrality target: Measuring the impact of electricity production from renewable energy sources and globalization. Journal of Environmental Management, 298. doi:10.1016/j.jenvman.2021.113460
Tiwari, S., Sharif, A., Nuta, F., Nuta, A. C., Cutcu, I., & Eren, M. V. (2023). Sustainable pathways for attaining net-zero emissions in European emerging countries - the nexus between renewable energy sources and ecological footprint. Environmental Science and Pollution Research, 16. doi:10.1007/s11356-023-29704-1
Wang, Y., Wang, D., & Shi, X. (2023). Sustainable development pathways of China's wind power industry under uncertainties: Perspective from economic benefits and technical potential. Energy Policy, 182. doi:10.1016/j.enpol.2023.113737
Xue, M., Lin, B. L., & Tsunemi, K. (2021). Emission implications of electric vehicles in Japan considering energy structure transition and penetration uncertainty. Journal of Cleaner Production, 280. doi:10.1016/j.jclepro.2020.124402
Xue, X., Zhang, Q., Cai, X., & Ponkratov, V. V. (2023). Multi-Criteria Decision Analysis for Evaluating the Effectiveness of Alternative Energy Sources in China. Sustainability (Switzerland), 15(10). doi:10.3390/su15108142
Yuan, K., Zhang, T., Xie, X., Du, S., Xue, X., Abdul-Manan, A. F. N., & Huang, Z. (2023). Exploration of low-cost green transition opportunities for China's power system under dual carbon goals. Journal of Cleaner Production, 414. doi:10.1016/j.jclepro.2023.137590
Zhai, H., Gu, B., Zhu, K., & Huang, C. (2023). Feasibility analysis of achieving net-zero emissions in China's power sector before 2050 based on ideal available pathways. Environmental Impact Assessment Review, 98, N.PAG-N.PAG. doi:10.1016/j.eiar.2022.106948
**Study type**
Benvenutti, L. M. M., & Campos, L. M. S. (2020). A fleet-based tank-to-wheel greenhouse gas emission analysis of light vehicles in Brazil and cooperation towards integrated policies. International Journal of Sustainable Transportation, 14(4), 255-269. doi:10.1080/15568318.2018.1542757
Gupta, R., Rüdisüli, M., Patel, M. K., & Parra, D. (2022). Smart power-to-gas deployment strategies informed by spatially explicit cost and value models. Applied Energy, 327. doi:10.1016/j.apenergy.2022.120015

Gupta, S., Kumar, R., & Kumar, A. (2024). Green hydrogen in India: Prioritization of its potential and viable renewable source. International Journal of Hydrogen Energy, 50, 226-238. doi:10.1016/j.ijhydene.2023.08.166
He, W., Abbas, Q., Alharthi, M., Mohsin, M., Hanif, I., Vinh Vo, X., & Taghizadeh-Hesary, F. (2020). Integration of renewable hydrogen in light-duty vehicle: Nexus between energy security and low carbon emission resources. International Journal of Hydrogen Energy, 45(51), 27958-27968. doi:10.1016/j.ijhydene.2020.06.177
Khan, M. Z. A., Khan, H. A., Ravi, S. S., Turner, J. W., & Aziz, M. (2023). Potential of clean liquid fuels in decarbonizing transportation – An overlooked net- zero pathway? Renewable and Sustainable Energy Reviews, 183. doi:10.1016/j.rser.2023.113483
Lau, H. C., & Tsai, S. C. (2022). A Decarbonization Roadmap for Taiwan and Its Energy Policy Implications. Sustainability (Switzerland), 14(14). doi:10.3390/su14148425
Li, K., Acha, S., Sunny, N., & Shah, N. (2022). Strategic transport fleet analysis of heavy goods vehicle technology for net-zero targets. Energy Policy, 168, N.PAG-N.PAG. doi:10.1016/j.enpol.2022.112988
Li, L., Wang, S., Zhang, S., Liu, D., & Ma, S. (2023). The Hydrogen Energy Infrastructure Location Selection Model: A Hybrid Fuzzy Decision-Making Approach. Sustainability (Switzerland), 15(13). doi:10.3390/su151310195
Minh Thong, L., Van Hiep, T., Thi Thu Thuy, B., & Huu Tung, D. (2021). The competition possibility between renewable energy and fossil energy in Vietnam in the future. Journal of World Energy Law and Business, 14(3), 215-228. doi:10.1093/jwelb/jwab021
Misila, P., Winyuchakrit, P., & Limmeechokchai, B. (2020). Thailand's long-term GHG emission reduction in 2050: the achievement of renewable energy and energy efficiency beyond the NDC. Heliyon, 6(12). doi:10.1016/j.heliyon.2020.e05720
Opoku-Mensah, E., Chun, W., Appiah-Otoo, I., Chen, W., & Tuffour, P. (2023). What level of renewable energy production will reduce ecological footprint without compromising trade? Evidence from Shanghai Cooperation Organization nations. Environmental science and pollution research international, 30(56), 119228-119242. doi:10.1007/s11356-023-30016-7
Palaniswamy, S., Sandhya Devi, R. S., Saravanan, M., & Anand, M. (2022). Social, Economic and Environmental Impact of Electric Vehicles in India. Journal of Environmental Science and Management, 25(1), 64-68. Retrieved from https://www.scopus.com/inward/record.uri?eid=2-s2.0-85134356555&partnerID=40&md5=cfc6a58a31010e62e093f5bcfcb46e40
Reddy, V. J., Hariram, N. P., Maity, R., Ghazali, M. F., & Kumarasamy, S. (2023). Sustainable E-Fuels: Green Hydrogen, Methanol and Ammonia for Carbon-Neutral Transportation. World Electric Vehicle Journal, 14(12). doi:10.3390/wevj14120349
Tan, K. M., Yong, J. Y., Ramachandaramurthy, V. K., Mansor, M., Teh, J., & Guerrero, J. M. (2023). Factors influencing global transportation electrification: Comparative analysis of electric and internal combustion engine vehicles. Renewable & Sustainable Energy Reviews, 184, N.PAG-N.PAG. doi:10.1016/j.rser.2023.113582
Tan, X., Tu, T., Gu, B., & Zeng, Y. (2021). Scenario simulation of CO2 emissions from light-duty passenger vehicles under land use-transport planning: A case of Shenzhen International Low Carbon City. Sustainable Cities and Society, 75. doi:10.1016/j.scs.2021.103266

Tarei, P. K., Chand, P., & Gupta, H. (2021). Barriers to the adoption of electric vehicles: Evidence from India. Journal of Cleaner Production, 291. doi:10.1016/j.jclepro.2021.125847
Wang, C. N., Nguyen, N. A. T., & Dang, T. T. (2022). Offshore wind power station (OWPS) site selection using a two-stage MCDM-based spherical fuzzy set approach. Scientific Reports, 12(1). doi:10.1038/s41598-022-08257-2
Wang, X., Zhou, Y., Bi, Q., Cao, Z., & Wang, B. (2022). Research on the Low-Carbon Development Path and Policy Options of China’s Transportation Under the Background of Dual Carbon Goals. Frontiers in Environmental Science, 10. doi:10.3389/fenvs.2022.905037
**Outcome**
Al-Nefaie, A. H., & Aldhyani, T. H. H. (2023). Predicting CO₂ Emissions from Traffic Vehicles for Sustainable and Smart Environment Using a Deep Learning Model. Sustainability, 15(9), 21. doi:10.3390/su15097615
Chang, C. C., Liao, Y. T., & Chang, Y. W. (2019). Life cycle assessment of alternative energy types – including hydrogen – for public city buses in Taiwan. International Journal of Hydrogen Energy, 44(33), 18472-18482. doi:10.1016/j.ijhydene.2019.05.073
García, I. C. G., Fernández-Guillamón, A., García-Cascales, M. S., & Molina-García, Á. (2024). Multi-factorial methodology for Wind Power Plant repowering optimization: A Spanish case study. Energy Reports, 11, 179-196. doi:10.1016/j.egyr.2023.11.044
Jani, H. K., Kachhwaha, S. S., Nagababu, G., Das, A., & Ehyaei, M. A. (2023). Energy, exergy, economic, environmental, advanced exergy and exergoeconomic (extended exergy) analysis of hybrid wind-solar power plant. Energy and Environment, 34(7), 2668-2704. doi:10.1177/0958305X221115095
Jeong, J., & Kim, H. (2021). DeepComp: Deep reinforcement learning based renewable energy error compensable forecasting. Applied Energy, 294. doi:10.1016/j.apenergy.2021.116970
Karipoğlu, F., Genç, M. S., & Koca, K. (2021). Determination of the most appropriate site selection of wind power plants based geographic information system and multi-criteria decision-making approach in develi, Turkey. International Journal of Sustainable Energy Planning and Management, 30, 97-114. doi:10.5278/ijsepm.6242
Kigle, S., Mohr, S., Kneiske, T., Clees, T., Ebner, M., Harper, R., . . . Ragwitz, M. (2024). TransHyDE-Sys: An Integrated Systemic Approach for Analyzing and Supporting the Transformation of Energy Systems and Hydrogen Infrastructure Development. Energy Technology. doi:10.1002/ente.202300828
Lindagato, P., Li, Y., Macháček, J., Yang, G., Mungwarakarama, I., Ndahimana, A., & Ntwali, H. P. K. (2023). Lithium Metal: The Key to Green Transportation. Applied Sciences (Switzerland), 13(1). doi:10.3390/app13010405
Lopez-Behar, D., Tran, M., Mayaud, J. R., Froese, T., Herrera, O. E., & Merida, W. (2019). Putting electric vehicles on the map: A policy agenda for residential charging infrastructure in Canada. Energy Research and Social Science, 50, 29-37. doi:10.1016/j.erss.2018.11.009
Moussavi, S., Barutha, P., & Dvorak, B. (2023). Environmental life cycle assessment of a novel offshore wind energy design project: A United States based case study. Renewable and Sustainable Energy Reviews, 185. doi:10.1016/j.rser.2023.113643

Mwandila, G., Mulenga, H., Thole, P., & Siwawa, E. (2024). Assessment of wind energy potential in Zambia. Energy for Sustainable Development, 78. doi:10.1016/j.esd.2024.101375
Parzen, M., Abdel-Khalek, H., Fedotova, E., Mahmood, M., Frysztacki, M. M., Hampp, J., . . . Fioriti, D. (2023). PyPSA-Earth. A new global open energy system optimization model demonstrated in Africa. Applied Energy, 341. doi:10.1016/j.apenergy.2023.121096
Quevedo, J., & Moya, I. H. (2022). Modeling of the dominican republic energy systems with OSeMOSYS to assess alternative scenarios for the expansion of renewable energy sources. Energy Nexus, 6. doi:10.1016/j.nexus.2022.100075
Sotiropoulou, K. F., & Vavatsikos, A. P. (2021). Onshore wind farms GIS-Assisted suitability analysis using PROMETHEE II. Energy Policy, 158. doi:10.1016/j.enpol.2021.112531
Vaiaso, T. V., & Jack, M. W. (2021). Quantifying the trade-off between percentage of renewable supply and affordability in Pacific island countries: Case study of Samoa. Renewable and Sustainable Energy Reviews, 150. doi:10.1016/j.rser.2021.111468
Vasebi, S., & Hayeri, Y. M. (2021). Collective Driving to Mitigate Climate Change: Collective-Adaptive Cruise Control. Sustainability, 13(16), 30. doi:10.3390/su13168943
**Population**
Hussain, B., Asif Ali Naqvi, S., Anwar, S., & Usman, M. (2023). Effect of wind and solar energy production, and economic development on the environmental quality: Is this the solution to climate change? Gondwana Research, 119, 27-44. doi:10.1016/j.gr.2023.01.012
Qing, L., Usman, M., Radulescu, M., & Haseeb, M. (2024). Towards the vision of going green in South Asian region: The role of technological innovations, renewable energy and natural resources in ecological footprint during globalization mode. Resources Policy, 88. doi:10.1016/j.resourpol.2023.104506

Reasons for exclusion are categorized under: intervention, outcome, study type and, population according to the eligibility criteria of the systematic review.

Supplementary Table 2. Policy recommendations.

**Study**	**Policy recommendation/s area focus**	**Policy recommendation/s outcome focus**	**Study policy recommendations** *(as stated by authors)*
Qadeer et al. 2023[1]	environmental	reduction of greenhouse gases emissions	*Hydrogen production is a significant step in ensuring its proper use, and it is also essential to understand that green hydrogen produced by electrolysis of water using renewable energy sources will succeed in achieving the Sustainable Development Goals (SDGs) (Arsad et al. 2022; Oliveira et al. 2021; Proost 2020). However, the Australian government should enact stringent regulations governing the production of green hydrogen by the electrolysis of water utilizing renewable energy sources. Alternatives that are more ecologically friendly should be used in place of obsolete and incompetent technology.*
Zhao et al. 2023[2]	energy safety	net zero target	*for the sake of stabilizing the GWTTN and contributing to the global vision of carbon neutrality, the key wind turbine trade countries should strengthen their mutual communication and minimize the risk of trade conflicts, such as the recent US–Sino trade war.*
Zhao et al. 2023[3]	environmental	climate change mitigation; net zero target	*governments in countries around the world should pay more attention to REC and generation in that renewable energy is more environmentally friendly than traditional fossil fuels….; Since REC and generation contribute to the mitigation of CLI, and geothermal energy has the most significant effect on CLI, the authorities should not only pay attention to the consumption of renewable energy but also promote the development of renewable energy generation, especially geothermal energy generation....; we should maximize the effect of REC on reducing society lock-in, and embed the promotion of clean and renewable energy into the education of residents and students.; policymakers should pay attention not only to renewable energy*

			but also to other measures, such as promoting the industrial structure and restricting the scale of thermal power plants.; Moreover, promoting a healthy lifestyle and increasing environmental consciousness is vital for reducing CLI in some developing countries. Furthermore, authorities in countries with a comparatively low level of CLI, such as European countries, should further increase the proportion of renewable energy by adopting tax reduction and exemption policies for REC and generation.; for some emerging economies, the energy market does not have strong financial support. The government should lower the investment threshold of renewable energy to create conditions for the formation of a clean and sustainable energy system.; trade tariffs on renewable energy exports and imports can be negotiated and reduced to promote more international imports and exports of renewable energy.; Technological innovations are found to improve environmental performance, and it is vital to increase the efficiency of renewable energy generation via more enhanced technology innovation. More environmentally related technological innovation can be developed and more funds can be used to support the exploration and exploitation of renewable energy.
Raihan et al. 2023[4]	environmental	reduction of greenhouse gases emissions	*In Thailand, the ultimate option for mitigating climate change is a low-carbon economy. To minimize contamination at its source, the "pollute first, then treat" policy and economic growth at the price of the environment might be modified. In this context, the study recommended that the government assist markets by establishing robust regulation that improves long-term emission reduction targets and continuously supports carbon- reducing technologies. Thailand's government can implement laws such as high carbon taxes, carbon capture and storage, and emission trading programs to limit CO2 emissions through fossil fuel use across electricity production and industries. Sectoral decoupling*

			necessitates considerable changes in centralized government policy, behavioral patterns, and scientific and technical development. Thailand may move its emphasis from extensive to intensive growth, altering its economic developmental trajectory by concentrating not only on productive capacity but also on sustainable economic improvement. Furthermore, fostering the economic switch to renewables is crucial for mitigating the environmental effects of economic expansion. Policymakers should also support and promote renewable energy technologies. These steps would stimulate economic growth and increase the share of renewable energy usage in final energy consumption by substituting CO2 -intensive traditional energy sources. Furthermore, organizational coordination is essential to promote renewable energy use across all economic operations to confirm long-term growth in the economy....; Long-term development in Thailand might be increased by adopting and executing efficient rules to manage the nation's industrial sector practices. The current analysis proposed that more clean energy or renewable energy be employed to enhance the energy utilization structure.,,,; Expanded use of renewable energy might have long-term effects on CO2 emissions and industrialization. Thailand could implement measures to decrease the price of renewable energy while discouraging the consumption of fossil fuels in industry, businesses, and families. Governmental policies to encourage renewable energy, and sustainable development may be implemented. The government would also encourage the use of energy-efficient residential equipment and more inexpensive renewable energies in the domestic sector. The government might formulate and maintain appropriate policies to encourage investment in the development of renewable energy technology, resulting in expanded renewable energy usage. The

			government, for example, would finance renewable energy projects through public–private partnerships. Furthermore, Thailand could be successful in establishing technical cooperation partnerships with developed countries whilst still researching renewable energy technology proactively. Additionally, local authorities and non-governmental groups can help to increase environmental awareness amongst persons of different ages by disseminating knowledge about green energy equipment, and energy efficiency. This could be achieved via training and instructional programs at schools and universities. Fiscal methods that the authorities may employ to motivate people to convert to cleaner energy include tax breaks, monetary assistance, and government contracts. The government may use media to spread its green living idea including low-carbon lifestyles and consumer behavior....; As a result, authorities may promote sustainable and green urbanization in order to reduce the possibility of environmental degradation, so increasing the role of renewables in following urbanization, such as the use of electric automobiles, solar lights, ethanol for vehicles, and so on. In urban regions, using renewable energy and energy-efficient devices and office machinery can help to minimize energy use and CO2 emissions....; Energy usage and production must be sustainable for green industrialization, particularly renewable energy use increase. Improving the share of renewable energy in total energy consumption will have a long-term influence on green industrialization....; Furthermore, the government could make it simpler for businesses to retain green and low-carbon technology and alternative energies for transport systems, logistics, accommodation, and other tourism-related activities, decreasing CO2 emissions and minimizing resource overexploitation...; To attain long-term agricultural output, the

			government may promote a more efficient power infrastructure and facilitate the transition to cleaner, more intensive agricultural energy sources...; government may encourage the usage of clean renewable energy such as wind, solar, and biofuel, as it increases agricultural output while simultaneously aiding in the fight against global warming and climate change. Incentives for the use of renewable energy in farming would help the firm compete in global markets while releasing fewer pollutants. Irrigation technologies can be shifted from non-renewable to renewable sources of energy to achieve a carbon–neutral environment.
Jahanger et al. 2023[5]	environmental/ energy	net zero target	*to assure environmental quality and sustainable growth and to significantly preserve carbon neutrality, policymakers in the top ten manufacturing nations must decrease investments in non-renewable energy sources and do the reverse, that is, by increasing investments in clean and sustained energy sources....; Again, environmental taxes and certification programmes for decarbonization are examples of policy alternatives that promote the use of green technologies in the manufacturing sector by measuring and rewarding progress toward efficiency goals. More specifically, these energy sources should come from renewable sources....; Furthermore, to reduce carbon emissions amongst the top ten manufacturing countries, these nations need to take advantage of developments in low-carbon electricity generation harnessing renewable energy sources; efforts from this and other sources will increase the frontier of energy efficiency used in production. Other decarbonization initiatives may include: utilizing cutting-edge emissions reductions technologies and strategies, such as carbon capture, usage, and storage, green hydrogen, powering processes with renewable electricity, using renewable sources (photovoltaic, solar thermal, or bio- based heat), increasing*

			energy efficiency across all industrial sectors by putting forward-thinking policies and technologies; and adopting a comprehensive strategy that goes beyond implementing standard industrial energy efficiency to decarbonize energy sources and other inputs to manufacturing applications in the areas of knowledge generation and dissemination on cutting-edge technologies and their relevance to the industrial sectors
Sun et al. 2023[6]	environmental	reduction of greenhouse gases emissions	*electic cars: the study proposes political ramifications for the five leading countries in increasing the commercial viability of BEVs and lowering CO2; At the market level, the five countries should highly urge improved transformation of the automobile sector. Tax incentives and subsidies on purchasing battery electric vehicles can encourage mass adoption, resulting in enormous benefits; strategic national policy endorsement and constant improvement of businesses in the renewable energy industry are prerequisites for increasing battery- powered vehicle sales.; Another critical factor in increasing demand for BEVs is increasing investment in charging stations.; Policymakers should also focus on raising consumer awareness about the benefits of adopting electric vehicles and decarbonizing power production. REC: governments can mitigate the negative environmental impact of energy consumption by progressively transitioning to 100% renewable energy consumption.; Larger organizations, for example automobile manufacturers, should establish and enforce laws to divert their portfolios to sustainable energies to replace fossil fuel usage, particularly in the USA and China, where oil and coal are the principal sources of primary energy. Furthermore, taxes on companies that consume large quantities of non-renewable energy such as coal should be increased, which can be used as a cross-subsidy to limit dependence on coal and expedite the pace of renewable energy transition. Again, for the countries to attain their CO2 emission objectives,*

			policy initiatives such as energy efficiency and investment in low urban areas should be a top priority.
Song et al. 2023[7]	energy	reduction of greenhouse gases emissions	*distributed power generation and grid integration technology using island renewable energy is essential to ensure a sustainable and stable power supply. Distributed generation uses distributed available energy to generate electricity, which can be directly connected to the distribution network or the user side. Compared with the traditional centralized power, distributed power generation is more flexible and reliable for the island environment (To et al., 2021), which is remote and with a small population. On this basis, the government should optimize the distribution network structure and improve the construction of a smart microgrid according to local resources. An off-grid micro-grid system integrating wind, light, storage, and load, and a grid-connected micro-grid system integrating light, storage and load are both necessary. The off-grid microgrid system adopts the self-use mode, while the grid-connected microgrid system can adopt the self-use and residual power grid connection mode. In addition, supporting energy storage systems can also help smooth the power curve and achieve peak and valley clipping.; To realize the energy-green transition of the island, governments need to not only increase the power generation from zero carbon sources, but also to control the energy demand (Climate Analytics, 2022). Local residents tend to consume more energy when they think that the energy produced by water and wind power is free. Thus, deepening the application of digital technologies in promoting the energy system transition based on the existing energy Internet is meaningful. Such approach to realizing refined energy management may help the government strengthen the guidance of residents and enterprises on energy conservation and carbon reduction. Relying on advanced communication and information*

			infrastructure, demand side management can be used to coordinate residential energy consumption and power generation from renewable energy changes (Pfeifer et al., 2018). In addition, vigorously developing ecotourism may aggravate the burden of local energy use and even break the island's relatively stable energy supply. Local governments must strictly regulate eco-friendly hotels, ecological facilities, and agricultural tourism projects to control the scale of tourists and strengthen the supervision of the efficiency and types of energy use in tourism (Ren et al., 2023b).
Gilmore et al. 2023[8]	environmental/ energy/ economic	reduction of greenhouse gases emissions	*Firstly, governments need to scale investment in zero emissions (renewably powered) technology by working with the domestic industry and OEMs to increase the deployment of very high penetration green hydrogen or biogas turbines. Secondly, governments could consider the introduction of a Green Gas Target modelled on Australia's existing Renewable Energy Target to drive investment in hydrogen production.*
Raihan et al. 2022[9]	environmental	reduction of greenhouse gases emissions	*Turkish environmental policy includes sustainable development through the renewable energy sector, particularly for wind and geothermal power that can aid Turkey in resolving its energy problem. Amidst this, technological, organizational, societal, governmental, and financial barriers have hampered the acquisition and utilization of sustainable resources. Turkey could implement measures to decrease the price of renewable energy while preventing the consumption of fossil fuels in industry, businesses, and families. The government would encourage the use of energy-efficient residential equipment and more inexpensive renewable energies in the domestic sector. The government might formulate and maintain appropriate policies to promote investment in the development of renewable energy technology, resulting in increased renewable energy usage. Furthermore, Turkey would be successful in establishing*

			technical cooperation partnerships with EU countries whilst still researching renewable energy technology proactively. The government may use media to spread its green living idea including low-carbon lifestyles and consumer behavior.
Raihan et al. 2022[10]	environmental	reduction of greenhouse gases emissions/ sustainable development	*Another suggestion is to re-examine current carbon tax laws while attempting to discover greener energy sources for Bangladesh's transportation industry and metropolitan areas.; Lower carbon emissions rates for electricity generation in Bangladesh would result from technological advancements in renewable energy integration. Thus, fostering the economic transition to renewables is critical for reducing the environmental pressures caused by economic development. Renewable energy companies and technology could also be encouraged and promoted by policymakers. By displacing CO2-intensive conventional energy sources, these measures will assist the economy in increasing the percentage of renewable energy consumption in overall energy consumption. In addition, institutional alignment is required to encourage renewable energy consumption across economic activities and assure long-term economic growth.; An increase in renewable energy usage would have a long-term impact on CO2 emissions and industrialization. Bangladesh's solar, hydro, wind, geothermal, and biomass energy resources can fully meet domestic energy demand. As a result, Bangladesh could benefit from international technical assistance networks and a rapid expansion of renewable energy sources. The government could devise and implement effective policies to promote investment in new renewable energy technologies, thereby increasing renewable energy consumption. For example, the government may invest in renewable energy projects through public-private partnerships. Renewable energy is abundant in Bangladesh, but promotion is hindered by higher costs. Bangladesh may develop*

			policies to reduce the cost of renewable energy and discourage the use of fossil fuels in industries, businesses, and households. Moreover, existing public education programs in Bangladesh on energy conservation and efficiency could be expanded. Regulatory policies may be established to promote renewable energy and environmental sustainability. The authorities would also promote energy-efficient home appliances and more affordable renewable energy sources for the household sector.
Raihan et al. 2022[11]	environmental	reduction of greenhouse gases emissions/ sustainable development	*To avoid pollution at the source, the "pollute first, then treat" strategy may be modified, as may the economic development mode at the price of the environment. In this context, we urge the government to support markets by developing a robust legislative framework that generates long-term value for emission reductions while also continuing to foster innovative technologies that drive to a less carbon-intensive economy.; Another suggestion is to re-examine current carbon tax laws while attempting to discover greener energy sources for Bangladesh's transportation industry and metropolitan areas.; Renewable energy companies and technology could also be encouraged and promoted by policymakers. By displacing CO2-intensive conventional energy sources, these measures will assist the economy in increasing the percentage of renewable energy consumption in overall energy consumption.; In addition, institutional alignment is essential to encourage the use of renewable energy across all economic activities and to ensure long-term economic prosperity.; An increase in renewable energy usage would have a long-term impact on CO2 emissions and industrialization. Bangladesh's solar, hydro, wind, geothermal, and biomass energy resources can fully meet domestic energy demand. As a result, Bangladesh could benefit from international technical assistance networks and a rapid expansion of renewable energy sources. The government could*

			devise and implement effective policies to promote investment in new renewable energy technologies, thereby increasing renewable energy consumption. For example, the government may capitalize on renewable energy projects through public-private partnerships. Bangladesh has plenty of renewable energy, but higher costs make promotion difficult. Bangladesh may develop policies to reduce the cost of renewable energy and discourage the use of fossil fuels in industries, businesses, and households. Moreover, existing public education programs in Bangladesh on energy conservation and efficiency could be expanded. Regulatory policies may be established to promote renewable energy and environmental sustainability. The authorities would also promote energy-efficient home appliances and more affordable renewable energy sources for the household sector.; Utilizing renewable energy and energy-efficiency appliances and office equipment can reduce energy consumption and CO2 emissions in metropolitan areas. Thus, sustainable urbanization and the popularization of renewable energy could be fostered by the government of Bangladesh.; The government may work hand in hand with local entities such as nongovernmental organizations (NGOs) and educational institutions to improve public awareness of renewable energy and a clean environment in metropolitan areas.; The study results suggest that government needs to invest more in public amenities and green technologies to enhance emission reduction in the urban region.;
Sun et al. 2022[12]	environmental/ energy/ economic	reduction of greenhouse gases emissions	*When considering the sources of CRI for clean energy, the overall approach should be focused on three perspectives: narrowing the technology gap between clean-energy power industries, narrowing the regional disparity between countries, and improving internal management. All countries and regions should continue to update and improve their clean-energy power*

			technology and equipment, strengthen technical assistance, and integrate power industry technology in a timely manner. At the same time, they should pay attention to non-technical work, such as institutional innovation, management innovation, talent training. Each country should adopt different policy priorities according to the source of its carbon emission reduction potential.; Asian, American, and European countries should *strengthen their information exchange and cooperation in the power generation industry between countries and regions, promote the effective dissemination of advanced power generation technologies and management experiences, reduce the renewable energy power generation technology gap between regions, and further promote the improvement of the CRE in the clean-energy power industry.*
Ifaei et al. 2022[13]	energy/ environment	sustainability	*Considering the positive correlation between WF and COE, the present wind-driven policies should be revised in favor of greater investment in biogas and solar energies. Wind farms can operate efficiently only on the western and eastern coasts, according to the generated hybrid maps. Hydrostatic energy was not found to be a barrier to sustainability.*
Horobet et al. 2022[14]	environmental	reduction of greenhouse gases emissions	*Through these findings we further contribute to the formulation of detailed approaches to tackle the relationship between energy sources and pollution to be included in environmental policies. Thus, we consider that additional efforts to build an energy mix that is less intensive in conventional fuels and oriented towards renewable and nuclear energy will lead to reduced environmental degradation and foster economic development. However, changes in the energy mix need to be adapted to countries' specific needs and resources endowments, optimizing the cost-effect component of the energy mix restructuring strategy.*<br>*Given the difficulty of decarbonizing transportation and heating,*

			clean electricity will become increasingly important, and we are seeing initiatives around the world aimed at electrifying various components of the energy system, such as shifting to electric vehicles, increasing heating system efficiency, or creating sustainable energy. In this context, requiring countries to benefit from readily available energy sources such as wind or Sun should become a global priority, particularly for developing economies. Furthermore, the increased share of renewable sources in electricity output in the electricity mix of many low- and middle-income countries is a good starting point for transforming their economic development process into an environmentally friendly one.
Raihan et al. 2022[15]	environmental	reduction of greenhouse gases emissions/ climate change mitigation	*To avoid pollution at the source, the "pollute first, then treat" strategy might be altered, and the economic development mode at the expense of the environment could be transformed.; lower carbon emissions rates for electricity generation in Peru would result from technological advancements in renewable energy integration. Thus, fostering the economic transition to renewables is critical for reducing the environmental pressures caused by economic development. Renewable energy companies and technology could also be encouraged and promoted by policymakers. By displacing CO2-intensive conventional energy sources, these measures would assist the economy in increasing the percentage of renewable energy consumption in overall energy consumption.; In order to reduce emissions, Peru might switch to renewable resources to take the place of traditional fuels. Increasing the share of renewable energy in overall energy usage would have a long-term impact on CO2 emissions reduction and industrialization. Peru is rich in solar energy, wind power resources, geothermal, and hydropower which can fully meet the domestic energy demand. Therefore, Peru could look forward to developing technical*

			assistance networks with other countries as well as vigorously developing their renewable resources. The government could create and execute effective support policies to encourage investment in new renewable energy technology to achieve a consistent and sustained increase in renewable energy consumption. The government might invest in renewable energy projects through public-private partnerships. Peru has tremendous renewable energy potential and already has a significant amount of renewable energy, but the higher cost of building and developing infrastructures for renewable energy technologies is preventing it from being promoted. Peru might develop measures to lower the cost of renewable energy and discourage the use of fossil fuels in industries, companies, and households since renewable energy use can help to reduce emissions. To raise public awareness of renewable energy and environmental sustainability, regulatory policies might be established. In addition, the authorities would concentrate on promoting energy-efficient residential electric appliances as well as more affordable renewable energy sources for the household sector.; The government might support the use of renewable energy, particularly clean renewable energy like solar and wind because it boosts agricultural productivity while also helping to battle global warming and climate change. Subsidies for renewable energy use in agriculture would help the industry become more competitive on worldwide markets while emitting less pollution.
Obobisa et al. 2022[16]	environmental	reduction of greenhouse gases emissions/ net zero target	*Renewable energy consumption reduces CO2 emissions in all four regions and the global level. The study recommends regulatory bodies incorporate renewable energy and energy efficiency concepts into their climate change plans, as well as in the design, implementation of regional and continental initiatives and programs to support the world transition to a more*

			sustainable and low-carbon energy world.; Governments should encourage public-private corporations through tax exemption and ecologically friendly budgets to promote the use of renewable energy appliances. Climate-minded policymakers should positively modify their energy mix and make policies based on renewable generation technologies such as wind and solar since it curbs CO2 emissions.; To reduce CO2 emissions and stabilize a rapidly warming climate, governments should encourage the use of clean energy, and increase investment in green technology. Countries should aim to eliminate the use of fossil fuels and transit to a low and zero-carbon world. Also, Climate-minded policymakers should implement unprecedented reforms and wean their citizens off fossil fuels by levying taxes on emissions-heavy imports and nonrenewable energy sources like oil, gas, and coal to avoid catastrophic levels of CO2 emissions.; Finally, to achieve 15 °C and net-zero emissions target, governments must continue to play a pivotal role in enabling innovators to pursue new ideas and supporting the best ones to find a route to profitability. Public funding for research and development is at the heart of this effort, and governments should increase it for clean energy technologies
Logan et al. 2020[17]	environmental	net zero target	*Push and pull TDM measures need to be implemented at a local and national level for policy makers to encourage the uptake of public transport and move away from private vehicles to decrease emissions.; Other measures for smaller towns including park and ride systems in conjunction with bus only zones within urban centres could be used. However, significant investment by the UK government and local authorities will need to be made for the infrastructure required for widespread integration of EBs and HBs into the mass transit system and to apply the TDM measures.; EBs produce the lowest level of emission from the three bus types studied and should be more*

			widely integrated into the public transport fleet.; Due to current technological advancements, EBs are more suited to shorter routes within cities such as London where as HBs have a larger range and may be suited to longer distances between cities and rural services. Therefore, integrating both bus types in favour of CFVs, EVs and CFBs would be more beneficial if the UK wants to meet their net zero targets.This, however, is dependent upon how the electricity is generated and stored. With a higher renewable and nuclear energy per- centage, the total level of operation emissions would be lower. HBs can be fuelled through electrolysis using excess renewable energy and with H2 generation from SMR using natural gas combined with CCS for low emissions.<br>*As technology currently isn't in place for 100% renewable electricity generation and gas generation will be required as dis-patchable power, CCS is required to reduce emissions as far as possible whilst infrastructure is being implemented to achieve complete decarbonisation of electricity generation.*
Govindarajan et al. 2021[18]	energy/ environment	decarbonization	*Also, the entry of private utility companies into the local RE market requires regulatory provisions to preserve competition and monitor fairness of electricity prices for distributed generation to be successful. In addition, there is a dearth of insufficient interconnection rules and technical feasibility for grid operations for any large-scale distribution generation (DG) scenarios for managing variability of RE. In some cases, policies add up energy storage costs for arriving at a final RE price, which then becomes substantially high. Also, RE prices affected by a global fall in photovoltaic module prices create doubts in the minds of customers.;cities can also plan large scale solar and wind energy projects such as solar parks and wind farms outside city limits and benefit from high yields to be supplied through successful banking, transmission and wheeling*

			arrangements. The necessary policies and regulations are required to be drafted to permit such successful banking of green energy at attractive tariffs for industrial consumers. In this regard, proper tariff designs and regulatory approvals embedding energy equity and justice principles are required for successful transition to a low carbon economy in cities.
Cheng et al. 2021[19]	technological innovation	reduction of greenhouse gases emissions	*First, due to the inconsistent impact of RETI on the carbon intensity reduction in the short and long term, the government should formulate differentiated policies from a short-term and long-term perspective respectively when promoting the development of RETI. The effect of RETI on environmental benefits is not evident in the short term, which illustrates that there are insufficient incentives for the development of RETI. Therefore, it is necessary to rely on the government intervention in the short run (e.g., demand-pull policies and technology-push policies) to correct the market failure of renewable energy technologies and realize the rapid advancement of renewable energy utilization. From the perspective of the long run, the impact of RETI on carbon intensity reduction is more obvious and significant. At this point, the government can rely on the market power to raise the proportion of renewable energy generation and put more effort into establishing the green energy consumption concept in the whole society.; Second, the gap between the innovation level of renewable energy technology in different regions of China is large, and the impact of RETI on the carbon intensity also shows the presence of heterogeneity. Therefore, in addition to the central government's guideline for renewable energy development, local governments need to further propose related incentives for technological innovation of renewable energy. The reason is that the impact on different provinces is not homogeneous, although the policies of the central government will affect the innovation levels of*

			renewable energy technologies in all provinces. Therefore, the local governments should draw up complementary policies according to the actual conditions, and better stimulate the innovative development of renewable energy. Although renewable energy technological innovations may exhibit spillover effects between different provinces in China, different provinces' abilities to introduce and absorb technological innovation have discrepancies due to the substantial differences in development levels. Generally speaking, the innovation level grows faster in economically developed regions because of more investment opportunities, advanced industrial structure, talents, and so on. It can also be seen from Fig. 1 that in 2000, the number of renewable energy patents in different regions was almost the same, and the next 15 years witness the innovation gaps quickly widen between provinces, especially between the eastern and western regions. Therefore, local governments should give full consideration to the abilities to introduce and absorb innovation when making decisions, and stimulate the utilization of renewable resources based on the different economic development status, environmental issues, and energy endowments.
Handayani et al. 2019[20]	energy/environment	reduction of greenhouse gases emissions	*Early deployment of renewable energy: With regard to timing, the deployment of renewable energy should start as early as possible to gain the benefits of technological learning. Moreover, the early deployment of renewable energy helps avoiding excessive investments in coal-based power plants and their related infrastructures, which have decades to serve after they are built.; Local learning: Conditions for future investments in renewable energy technologies in developing countries depend on a combination of global and local learning processes. Since local learning has a significant impact on the costs of renewable energy (Huenteler et al., 2016), the conditions that enable faster*

			local learning should be made available. These include an increased number of skilled workforce, a stable regulatory framework, and the establishment of sustainable business models. Furthermore, improvement in infrastructure, such as accessibility of remote areas, is required to enable faster distribution of renewable energy technologies. Moreover, the involvement of all parties, including users, suppliers, competitors, universities, and regulators, is critical as interactions between them is the key for the learning and innovation processes to occur (Lundvall, 2016).; Improvements in the grid capacity: Integration of the vast renewable energy capacity presents new challenges to any electricity system operations and planning. Variable energy resources, such as wind and solar, have intermittent characteristics, which will likely change the way electricity is dispatched and transmitted by the grid operator. Therefore, the acceleration of renewable energy deployment should go hand-in-hand with the improvement of grid capacity in terms of technical and human capital capacity. Furthermore, other disruptive technologies, such as the internet of energy, energy storage, and electric vehicles, require the global utility sector for transforming to a smarter grid.
Cheng et al. 2019[21]	environmental	reduction of greenhouse gases emissions	*development of renewable energy. Although the process of industrialization needs plenty of natural resources, especially energy, the BRIICS countries can accelerate the development of renewable energy. The development of renewable energy can not only satisfy the energy need of the industrialization, but also mitigate carbon emissions.*
Calvillo et al. 2020[22]	environmental	net zero target/ climate change mitigation	*The results obtained show the importance of the 'smartness' and location of EV charging for network reinforcements costs. Also, network investment costs are passed to the final consumers as an increase in energy marginal costs (energy prices). Therefore, a charging scheme with higher network*

			investment and more expensive electricity generation will translate to higher energy bills to final consumers. This will not only affect the costs of charging EVs, but of all electricity-powered services across the energy system as well. This could create a cascading effect, increasing prices in other non-energy goods and services. These cost increases are an important burden particularly for those in lower incomes, who might not even benefit from having an EV. It is, therefore important to consider these outcomes while designing energy tariffs and EV policies, making sure that there is a reasonable balance of costs and benefits across the whole economy.; Looking into CO2 emissions, all EV scenarios presented a similar reduction in emissions for the transport sector. However, we observe a shift of sectoral emissions as the power sector increased their emissions (extra generation to meet EV demand), effectively reducing the potential climate benefits of the introduction of EVs. These results show the importance of a whole system approach to tackle climate change, where there is no emission transfer to other sectors or 'outsourced' to other countries.; The type of study proposed in this paper provides valuable insight on the implications on network investments and energy costs of different types of EV charging options. Moreover, this study brings other important points of discussion on the EV rollout, including the timing of network investments, the economic impacts to consumers (direct and indirect), and the potential benefits to the wider economy. Therefore, we see this analysis as necessary first step for further research on the full implications of the EV rollout in the energy system and the wider economy.
Hossain et al. 2023[23]	environmental	sustainability	*Nuclear energy is a strong candidate as an alternate for fossil fuels as clean and green energy since it offers low carbon emissions, and competitiveness. However, renewable energies*

			such as solar, wind, hydroelectric, biomass are virtually inexhaustible in duration in addition to being clean and green energy. Although increasing the use of renewable energy may not bring immediate positive results, it would facilitate reaching environmental sustainability in the long-run. Therefore, the USA should take long-term energy policy while diversifying its energy mix to achieve environmental sustainability by adopting renewable and nuclear energy sources and reducing the dependency on fossil fuel energy. To increase the share of green energy in the mix, policymakers can reduce the demand for fossil fuel energy by increasing the energy price or increasing the carbon tax rate to the producers or reducing the subsidy. On the contrary, adequate incentives can increase the use of renewable and nuclear energy. Fortunately, the Renewable Portfolio Standard has been introduced in several states around the USA to encourage the expansion of renewable energy. However, this policy can be implemented throughout all states to achieve greater reductions in emissions. In order to improve energy efficiency, the government must also expand investment in nuclear and renewable energy infrastructure.

All policy recommendations are taken as direct quotes from the papers. Only the policy recommendations that are relevant to this systematic review were extracted and reported.

Supplementary Table 3. Evidence Communication Rules for Policy (ECR-P) critical appraisal tool

		response options and judgement
	Domain and signalling question	low risk of bias/ some concerns/ high risk of bias
**Level**	**Domain 1: Inform not persuade**	
**study**	1.1 Were the aims/objectives for the study defined?	Y/PY/N/PN/NI
	1.2 Were the limitations of the study findings reported?	Y/PY/N/PN/NI
	If Y/PY to 1.2:<br>1.2.1 Did the study propose ways to reduce limitations in the future?	Y/PY/N/PN/NI/NA
	1.3 Were the study conclusions clearly connected to the findings of the study?	Y/PY/N/PN/NI
	1.4 Was emotive language avoided in communicating study findings and/or conclusions?	Y/PY/N/PN/NI
**policy recommendations**	1.5 Were the aims/objectives for the policy recommendations defined?	Y/PY/N/PN/NI
	1.6 Were the limitations of the policy recommendations reported?	Y/PY/N/PN/NI
	1.7 Were the policy recommendations clearly connected to the findings of the study?	Y/PY/N/PN/NI
	1.8 Was accessible language used for the policy recommendations?	Y/PY/N/PN/NI
	1.9 Was emotive language avoided in policy recommendations?	Y/PY/N/PN/NI
	**Domain 2: Offer balance, not false balance**	
**study**	2.1 Were all aspects of the study findings reported?	Y/PY/N/PN/NI
	2.2 Was an appropriate reporting guideline used for constructing the manuscript?	Y/PY/N/PN/NI
**policy recommendations**	2.3 Were multiple implications of the policy recommendations considered?	Y/PY/N/PN/NI
	2.4 Was the existence of a current policy discussed?	Y/PY/N/PN/NI
	If Y/PY to 2.4<br>2.4.1 Was not changing the current policy considered?	Y/PY/N/PN/NI/NA
	**Domain 3: Disclose uncertainties**	

**study**	3.1. Were uncertainties of the study findings reported?	Y/PY/N/PN/NI
	If Y/PY to 3.1<br>3.1.1 Did the study propose ways to reduce uncertainties in the future?	Y/PY/N/PN/NI/NA
**policy recommendations**	3.2 Were uncertainties of the policy recommendations reported?	Y/PY/N/PN/NI
	If Y/PY to 3.2<br>3.2.1 Did the study adopt a precautionary principle perspective?	Y/PY/N/PN/NI/NA
	**Domain 4: State evidence quality**	
**study**	4.1 Was the quality of the evidence used in the analysis considered?	Y/PY/N/PN/NI
	If Y/PY to 4.1<br>4.1.1 Were specific metrics of evidence quality used?	Y/PY/N/PN/NI/NA
**policy recommendations**	4.2 Was the quality of the study findings, that formulated the evidence base for the policy recommendations, considered?	Y/PY/N/PN/NI
	**Domain 5: Pre-empt misunderstandings**	
**study**	5.1 Were potential misunderstandings about the study findings and conclusions pre-emptively addressed?	Y/PY/N/PN/NI
**policy recommendations**	5.2 Was the targeted audience for policy recommendations defined?	Y/PY/N/PN/NI
	5.3 Were potential misunderstandings for policy recommendations and potential concerns of the policy makers pre-emptively addressed?	Y/PY/N/PN/NI

Supplementary Table 4. Risk of bias ratings for Collaboration for Environmental Evidence critical appraisal tool (CEECAT) and Evidence Communication Rules for Policy (ECR-P) critical appraisal tool.

	Risk of Bias rating			
	**CEECAT**	**ECP-R**		
**Study**	Overall	Study level	PR level	Overall
Calvillo and Turner 2020[22]	medium	high	high	high
Cheng and Yao 2021[19]	high	high	high	high
Cheng et al. 2019[21]	high	high	high	high
Gilmore et al. 2023[8]	medium	high	high	high
Govindarajan et al. 2021[18]	high	high	high	high
Handayani et al. 2019[20]	medium	medium	high	high
Horobet et al. 2022[14]	high	high	high	high
Hossain et al. 2023[23]	medium	high	high	high
Ifaei et al. 2022[13]	medium	high	high	high
Jahanger et al. 2023[5]	high	medium	high	high
Logan et al. 2020[17]	medium	medium	high	high
Obobisa et al. 2022[16]	high	high	high	high
Qadeer et al. 2023[1]	high	medium	high	high
Raihan and Tuspekova 2022a[15]	high	high	high	high
Raihan and Tuspekova 2022b[9]	high	high	high	high
Raihan et al. 2022a[11]	high	high	high	high
Raihan et al. 2022b[10]	high	high	high	high
Raihan et al. 2023[4]	high	high	high	high
Song and Chen 2023[7]	high	high	high	high
Sun and Dong 2022[12]	medium	high	high	high
Sun et al. 2023[6]	medium	high	high	high
Zhao C. et al. 2023[3]	low	high	high	high
Zhao L. et al. 2023[2]	high	high	high	high

Green cells denote agreement between overall rating between the two tools. Purple denotes agreement between the overall rating of CEECAT the level rating for ECP-R. ‘medium’ rating stands for ‘some concerns’ for ECP-R.

Supplementary Table 5. PRISMA 2020 Checklist[24]

Section and Topic	Item #	Checklist item	Location where item is reported
**TITLE**			
Title	1	Identify the report as a systematic review.	p. 1
**ABSTRACT**			
Abstract	2	See the PRISMA 2020 for Abstracts checklist.	p. 2
**INTRODUCTION**			
Rationale	3	Describe the rationale for the review in the context of existing knowledge.	p. 3-4
Objectives	4	Provide an explicit statement of the objective(s) or question(s) the review addresses.	p. 5
**METHODS**			
Eligibility criteria	5	Specify the inclusion and exclusion criteria for the review and how studies were grouped for the syntheses.	p. 23-24
Information sources	6	Specify all databases, registers, websites, organisations, reference lists and other sources searched or consulted to identify studies. Specify the date when each source was last searched or consulted.	p. 24-25
Search strategy	7	Present the full search strategies for all databases, registers and websites, including any filters and limits used.	Supp. information
Selection process	8	Specify the methods used to decide whether a study met the inclusion criteria of the review, including how many reviewers screened each record and each report retrieved, whether they worked independently, and if applicable, details of automation tools used in the process.	p. 24-25
Data collection process	9	Specify the methods used to collect data from reports, including how many reviewers collected data from each report, whether they worked independently, any processes for obtaining or confirming data from study investigators, and if applicable, details of automation tools used in the process.	p. 24-25
Data items	10a	List and define all outcomes for which data were sought. Specify whether all results that were compatible with each outcome domain in each study were sought (e.g. for all measures, time points, analyses), and if not, the methods used to decide which results to collect.	p. 24-25
	10b	List and define all other variables for which data were sought (e.g. participant and intervention characteristics, funding sources). Describe any assumptions made about any missing or unclear information.	p. 24-25
Study risk of bias assessment	11	Specify the methods used to assess risk of bias in the included studies, including details of the tool(s) used, how many reviewers assessed each study and whether they worked independently, and if applicable, details of automation tools used in the process.	p. 25-27
Effect measures	12	Specify for each outcome the effect measure(s) (e.g. risk ratio, mean difference) used in the synthesis or presentation of results.	Not applicable
Synthesis methods	13a	Describe the processes used to decide which studies were eligible for each synthesis (e.g. tabulating the study intervention characteristics and comparing against the planned groups for each synthesis (item #5)).	p. 24-25
	13b	Describe any methods required to prepare the data for presentation or synthesis, such as handling of missing summary statistics, or data conversions.	p. 24-25

Section and Topic	Item #	Checklist item	Location where item is reported
	13c	Describe any methods used to tabulate or visually display results of individual studies and syntheses.	p. 25
	13d	Describe any methods used to synthesize results and provide a rationale for the choice(s). If meta-analysis was performed, describe the model(s), method(s) to identify the presence and extent of statistical heterogeneity, and software package(s) used.	p. 25
	13e	Describe any methods used to explore possible causes of heterogeneity among study results (e.g. subgroup analysis, meta-regression).	Not applicable
	13f	Describe any sensitivity analyses conducted to assess robustness of the synthesized results.	Not applicable
Reporting bias assessment	14	Describe any methods used to assess risk of bias due to missing results in a synthesis (arising from reporting biases).	p. 27-28
Certainty assessment	15	Describe any methods used to assess certainty (or confidence) in the body of evidence for an outcome.	p. 27-28
**RESULTS**			
Study selection	16a	Describe the results of the search and selection process, from the number of records identified in the search to the number of studies included in the review, ideally using a flow diagram.	p. 5-6, Figure 1
	16b	Cite studies that might appear to meet the inclusion criteria, but which were excluded, and explain why they were excluded.	Figure 1, Sup. Table 1
Study characteristics	17	Cite each included study and present its characteristics.	Table 1, p.7
Risk of bias in studies	18	Present assessments of risk of bias for each included study.	p. 7-15, Figures 2-5, Sup. Figures 2-5
Results of individual studies	19	For all outcomes, present, for each study: (a) summary statistics for each group (where appropriate) and (b) an effect estimate and its precision (e.g. confidence/credible interval), ideally using structured tables or plots.	p. 7
Results of syntheses	20a	For each synthesis, briefly summarise the characteristics and risk of bias among contributing studies.	p. 13-15
	20b	Present results of all statistical syntheses conducted. If meta-analysis was done, present for each the summary estimate and its precision (e.g. confidence/credible interval) and measures of statistical heterogeneity. If comparing groups, describe the direction of the effect.	Not applicable
	20c	Present results of all investigations of possible causes of heterogeneity among study results.	Not applicable
	20d	Present results of all sensitivity analyses conducted to assess the robustness of the synthesized results.	Not applicable

Section and Topic	Item #	Checklist item	Location where item is reported
Reporting biases	21	Present assessments of risk of bias due to missing results (arising from reporting biases) for each synthesis assessed.	Not applicable
Certainty of evidence	22	Present assessments of certainty (or confidence) in the body of evidence for each outcome assessed.	p. 13-15
**DISCUSSION**			
Discussion	23a	Provide a general interpretation of the results in the context of other evidence.	p. 15
	23b	Discuss any limitations of the evidence included in the review.	p. 20
	23c	Discuss any limitations of the review processes used.	p. 17
	23d	Discuss implications of the results for practice, policy, and future research.	p. 21-22
**OTHER INFORMATION**			
Registration and protocol	24a	Provide registration information for the review, including register name and registration number, or state that the review was not registered.	p. 22
	24b	Indicate where the review protocol can be accessed, or state that a protocol was not prepared.	p. 22
	24c	Describe and explain any amendments to information provided at registration or in the protocol.	Not applicable
Support	25	Describe sources of financial or non-financial support for the review, and the role of the funders or sponsors in the review.	p. 29
Competing interests	26	Declare any competing interests of review authors.	p. 29
Availability of data, code and other materials	27	Report which of the following are publicly available and where they can be found: template data collection forms; data extracted from included studies; data used for all analyses; analytic code; any other materials used in the review.	p. 29

Supplementary Table 6. Web of Science search strategy

1	"(TS=(net zero)) OR TS=(climate change)"
2	"(((((AB=(recommendation*)) OR AB=(implication*)) OR AB=(making)) OR AB=(suggestion*)) OR AB=(maker*)) AND AB=(policy OR policies)"
3	"power (Topic) OR energy (Topic)"
4	"wind (Topic) OR hydrogen (Topic)"
5	"#3 AND #4"
6	"#1 AND #2 AND #5"
7	"TS=(renewable OR sustainable OR green)"
8	"TS=(transport*) OR TS=(energ*) OR TS=(fuel*) OR TS=(power*)"
9	"#7 AND #8"
10	"#1 AND #9 AND #2"
11	"#6 OR #10"

Supplementary Figure 1. Thematic analysis of policy recommendations

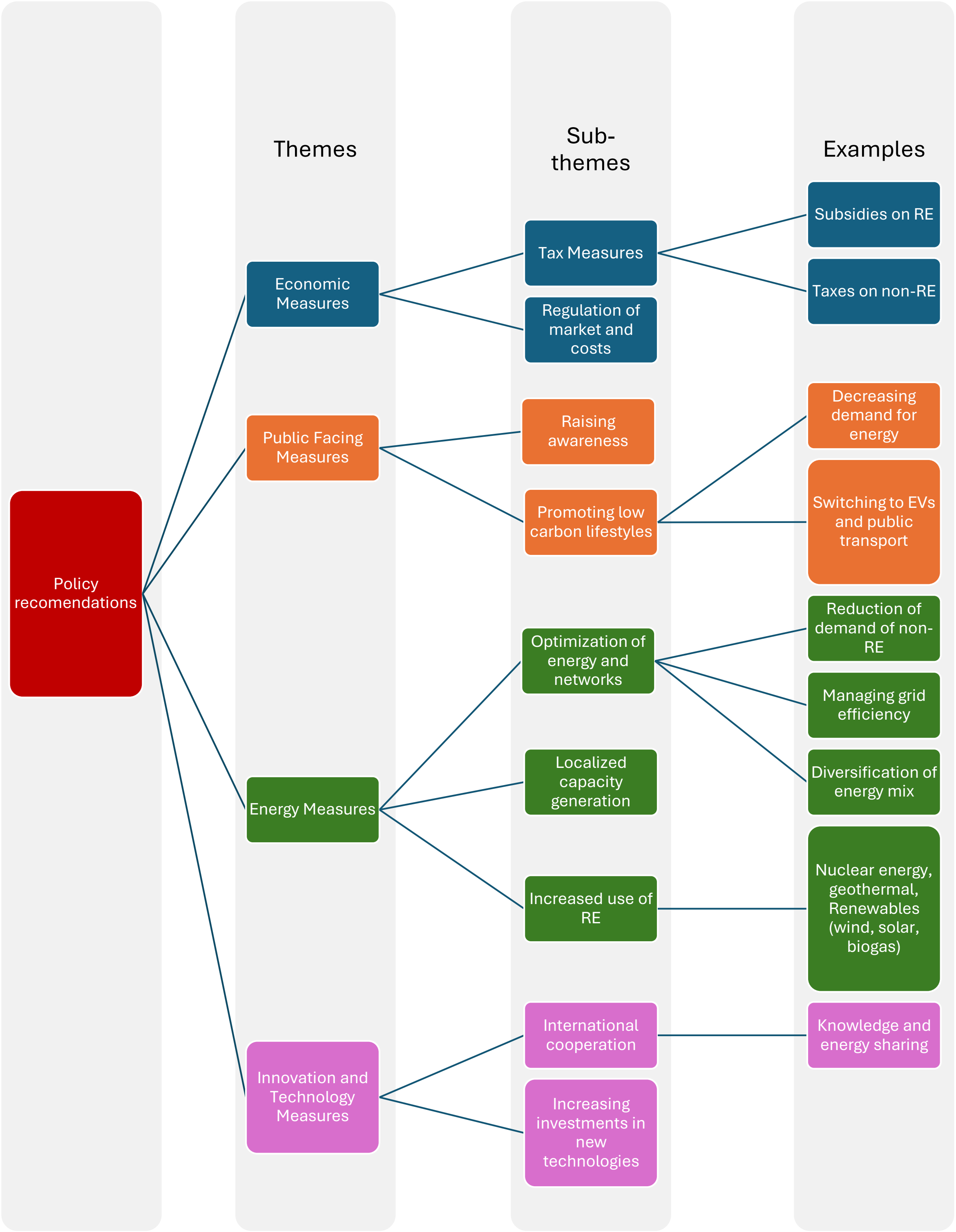

RE, renewable energy/ies

Supplementary Figure 2. Evidence Communication Rules for Policy (ECR-P) critical appraisal tool summary of results for study level.

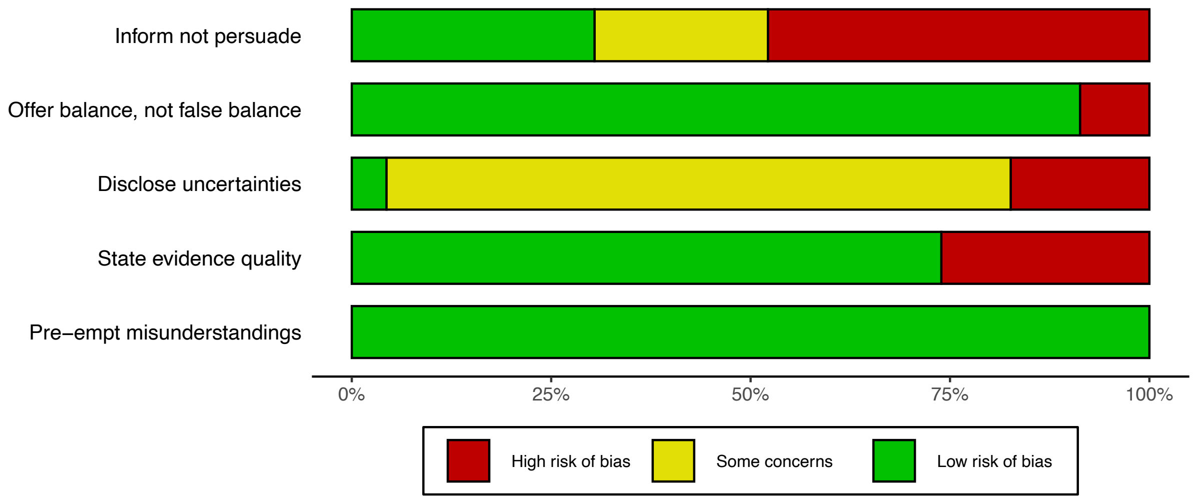

The Evidence Communication Rules for Policy (ECR-P) critical appraisal tool addresses the risk of bias (RoB) in the five different domains illustrated in the five top bars of the figure. An overall RoB rating is also attributed to each paper. Here, the three ratings of high RoB, some concerns and low RoB are illustrated by percentage for the entirety of the 23 studies that are included in the systematic review. Ratings refer only to the study level section of the tool.

Supplementary Figure 3. Evidence Communication Rules for Policy (ECR-P) critical appraisal tool summary of results for policy recommendations level.

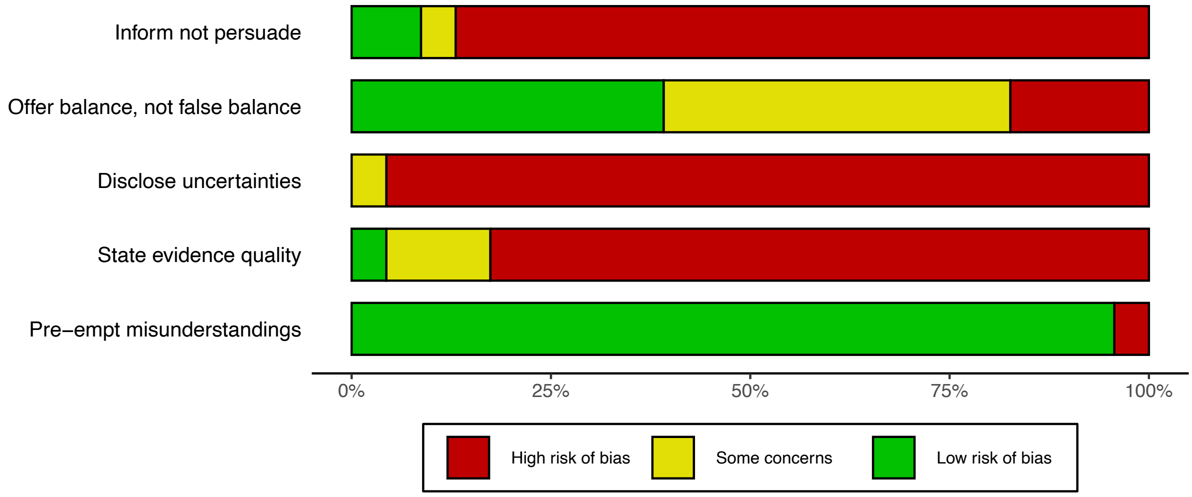

The Evidence Communication Rules for Policy (ECR-P) critical appraisal tool addresses the risk of bias (RoB) in the five different domains illustrated in the five top bars of the figure. An overall RoB rating is also attributed to each paper. Here, the three ratings of high RoB, some concerns and low RoB are illustrated by percentage for the entirety of the 23 studies that are included in the systematic review. Ratings refer only to the policy recommendations level section of the tool.

Supplementary Figure 4. Evidence Communication Rules for Policy (ECR-P) critical appraisal tool individual study results for study level.

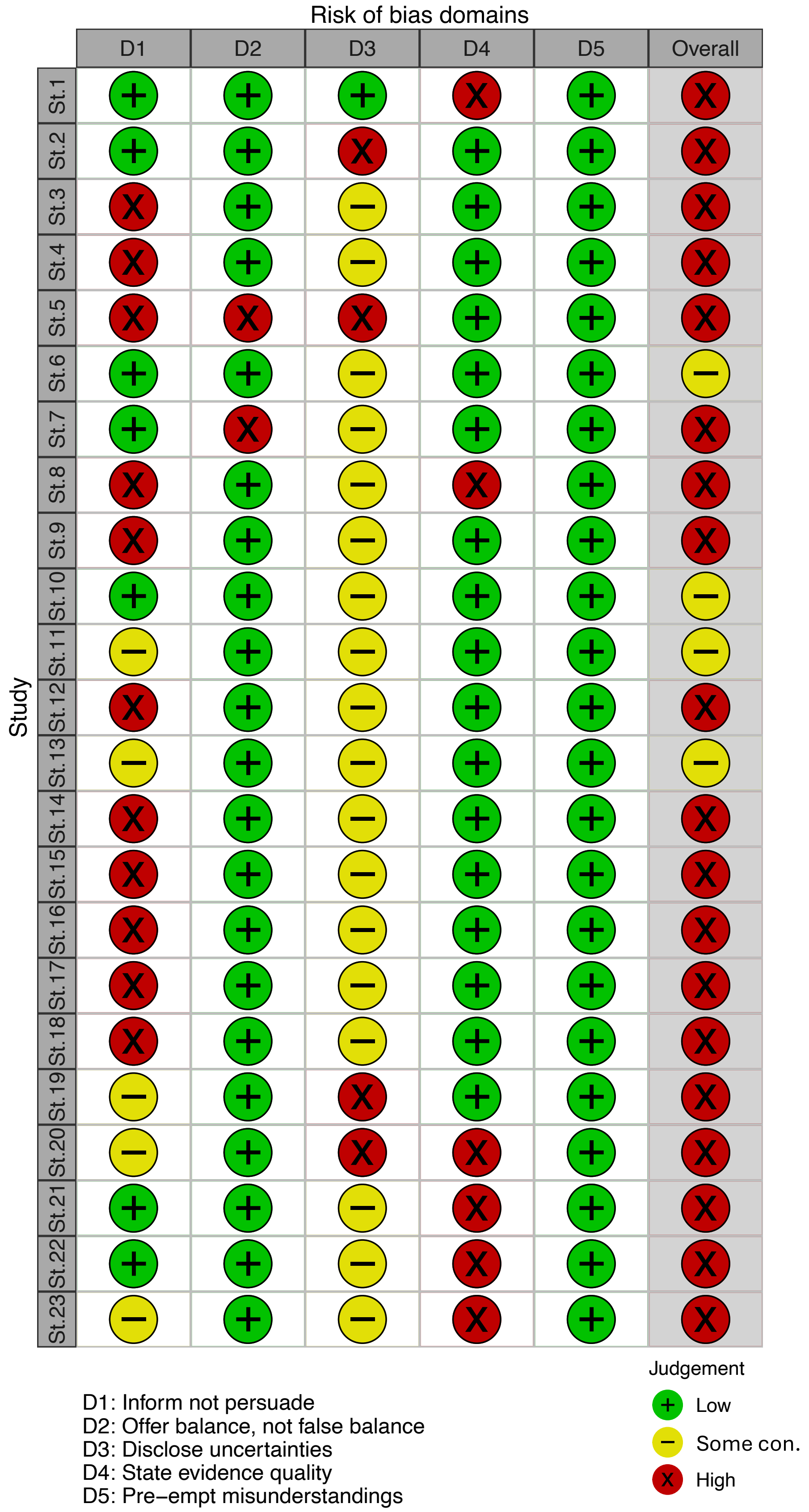

The Evidence Communication Rules for Policy (ECR-P) critical appraisal tool addresses the risk of bias (RoB) in five different domains; D1: Inform not persuade, D2: Offer balance, not false balance, D3: Disclose uncertainties, D4: State evidence quality, D5: pre-empt misunderstandings. (x) indicates high RoB, (+) indicates low RoB and (-) indicates some concerns. Ratings refer only to the study level section of the tool. RoB is examined and presented for each individual study: St.1, Calvillo and Turner (2020)[22]; St.2, Cheng and Yao (2021)[19]; St.3, Cheng et al. (2019)[21]; St.4, Gilmore et al. (2023)[8]; St.5, Govindarajan and Ganesh (2021)[18]; St.6, Handayani et al. (2019)[20]; St.7, Horobet et al. (2022)[14]; St.8, Hossain et al. (2023)[23]; St.9, Ifaei et al. (2022)[13]; St.10, Jahanger et al. (2023)[5]; St.11, Logan et al. (2020)[17]; St.12, Obobisa (2022)[16]; St.13, Qadeer et al. (2023)[1]; St.14, Raihan and Tuspekova (2022b)[15]; St.15, Raihan and Tuspekova (2022a)[9]; St.16, Raihan et al. (2022a)[11]; St.17, Raihan et al. (2022b)[10]; St.18, Raihan et al. (2023)[4]; St.19, Song and Chen (2023)[7]; St.20, Sun and Dong (2022)[12]; St.21, Sun et al. (2023)[6]; St.22, Zhao, C. et al. (2023)[3]; St.23, Zhao, L. et al. (2023)[2]

Supplementary Figure 5. Evidence Communication Rules for Policy (ECR-P) critical appraisal tool individual study results for policy recommendations level.

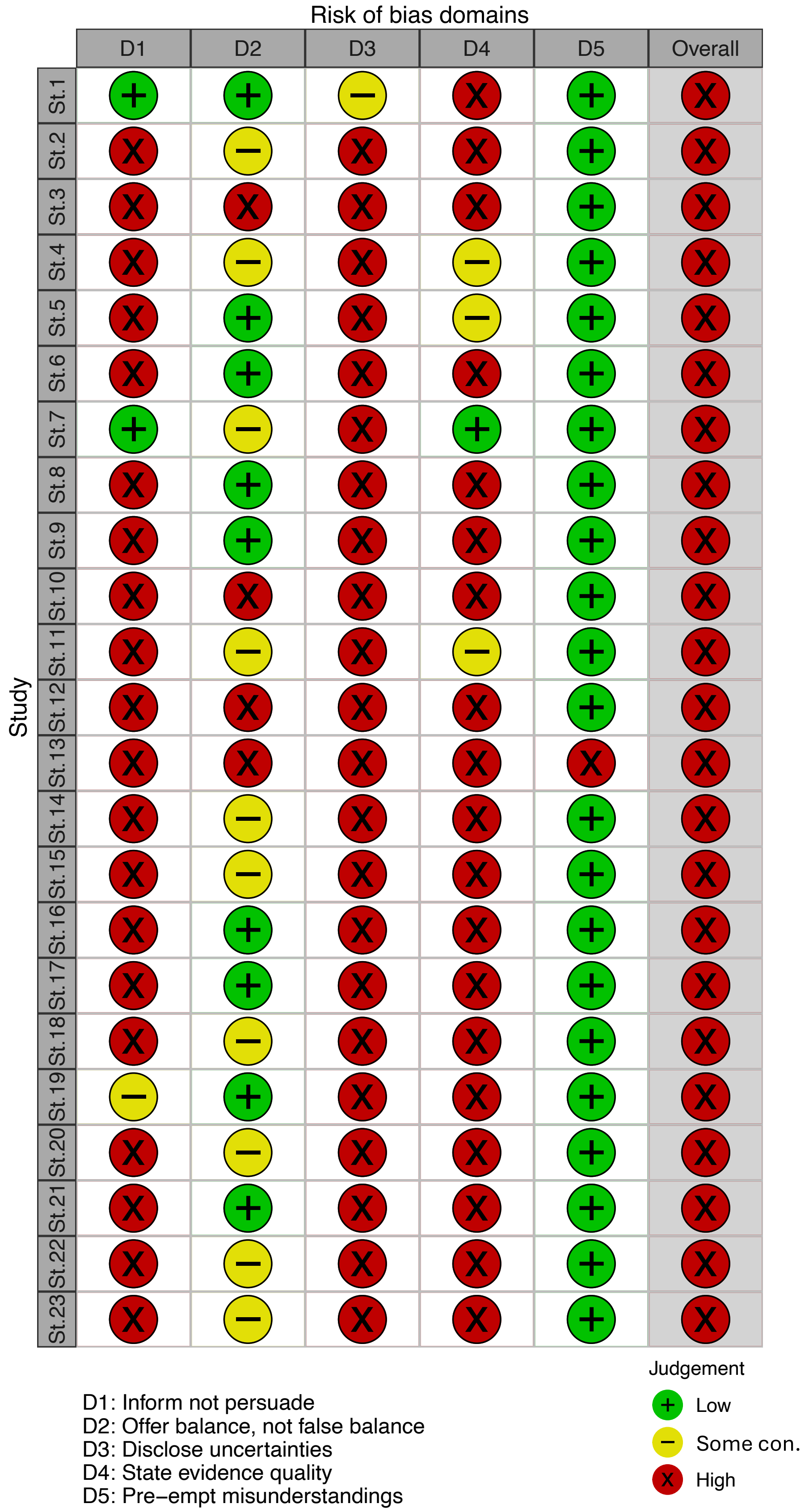

The Evidence Communication Rules for Policy (ECR-P) critical appraisal tool addresses the risk of bias (RoB) in five different domains; D1: Inform not persuade, D2: Offer balance, not false balance, D3: Disclose uncertainties, D4: State evidence quality, D5: pre-empt misunderstandings. (x) indicates high RoB, (+) indicates low RoB and (-) indicates some concerns. Ratings refer only to the policy recommendations level section of the tool. RoB is examined and presented for each individual study: St.1, Calvillo and Turner (2020)[22]; St.2, Cheng and Yao (2021)[19]; St.3, Cheng et al. (2019)[21]; St.4, Gilmore et al. (2023)[8]; St.5, Govindarajan and Ganesh (2021)[18]; St.6, Handayani et al. (2019)[20]; St.7, Horobet et al. (2022)[14]; St.8, Hossain et al. (2023)[23]; St.9, Ifaei et al. (2022)[13]; St.10, Jahanger et al. (2023)[5]; St.11, Logan et al. (2020)[17]; St.12, Obobisa (2022)[16]; St.13, Qadeer et al. (2023)[1]; St.14, Raihan and Tuspekova (2022b)[15]; St.15, Raihan and Tuspekova (2022a)[9]; St.16, Raihan et al. (2022a)[11]; St.17, Raihan et al. (2022b)[10]; St.18, Raihan et al. (2023)[4]; St.19, Song and Chen (2023)[7]; St.20, Sun and Dong (2022)[12]; St.21, Sun et al. (2023)[6]; St.22, Zhao, C. et al. (2023)[3]; St.23, Zhao, L. et al. (2023)[2]

References

1 Qadeer, A., Hussan, M. W., Aziz, G., Waheed, R. & Sarwar, S. Emerging trends of green hydrogen and sustainable environment in the case of Australia. *Environ Sci Pollut Res Int* **30**, 115788-115804 (2023). https://doi.org/10.1007/s11356-023-30560-2

2 Zhao, L. *et al.* Structure, robustness and supply risk in the global wind turbine trade network. *Renewable Sustainable Energy Rev* **177** (2023). https://doi.org/10.1016/j.rser.2023.113214

3 Zhao, C., Wang, J., Dong, K. & Wang, K. How does renewable energy encourage carbon unlocking? A global case for decarbonization. *Resour. Policy* **83** (2023). https://doi.org/10.1016/j.resourpol.2023.103622

4 Raihan, A. *et al.* Dynamic Linkages between Environmental Factors and Carbon Emissions in Thailand. *Environ. Process.* **10** (2023). https://doi.org/10.1007/s40710-023-00618-x

5 Jahanger, A., Ozturk, I., Chukwuma Onwe, J., Joseph, T. E. & Razib Hossain, M. Do technology and renewable energy contribute to energy efficiency and carbon neutrality? Evidence from top ten manufacturing countries. *Sustainable Energy Technol. Assess.* **56** (2023). https://doi.org/10.1016/j.seta.2023.103084

6 Sun, D., Kyere, F., Sampene, A. K., Asante, D. & Kumah, N. Y. G. An investigation on the role of electric vehicles in alleviating environmental pollution: evidence from five leading economies. *Environ. Sci. Pollut. Res.* **30**, 18244-18259 (2023). https://doi.org/10.1007/s11356-022-23386-x

7 Song, X. & Chen, Z. Pathways for an island energy transition under climate change: The case of Chongming Island, China. *Front. Energy Res.* **11** (2023). https://doi.org/10.3389/fenrg.2023.1126411

8 Gilmore, J., Nelson, T. & Nolan, T. Firming Technologies to Reach 100% Renewable Energy Production in Australia's National Electricity Market (NEM). *Energy J.* **44**, 189-210 (2023). https://doi.org/10.5547/01956574.44.6.jgil

9 Raihan, A. & Tuspekova, A. Dynamic impacts of economic growth, renewable energy use, urbanization, industrialization, tourism, agriculture, and forests on carbon emissions in Turkey. *Carbon. Res.* **1** (2022). https://doi.org/10.1007/s44246-022-00019-z

10 Raihan, A., Muhtasim, D. A., Khan, M. N. A., Pavel, M. I. & Faruk, O. Nexus between carbon emissions, economic growth, renewable energy use, and technological innovation towards achieving environmental sustainability in Bangladesh. *Clean. Energy. Syst.* **3** (2022). https://doi.org/10.1016/j.cles.2022.100032

11 Raihan, A. *et al.* Nexus between carbon emissions, economic growth, renewable energy use, urbanization, industrialization, technological innovation, and forest area towards achieving environmental sustainability in Bangladesh. *Energy Clim. Chang.* **3** (2022). https://doi.org/10.1016/j.egycc.2022.100080

12 Sun, J. & Dong, F. Decomposition of carbon emission reduction efficiency and potential for clean energy power: Evidence from 58 countries. *J. Clean. Prod.* **363** (2022). https://doi.org/10.1016/j.jclepro.2022.132312

13 Ifaei, P., Tayerani Charmchi, A. S., Loy-Benitez, J., Yang, R. J. & Yoo, C. A data-driven analytical roadmap to a sustainable 2030 in South Korea based on optimal renewable microgrids. *Renewable Sustainable Energy Rev* **167** (2022). https://doi.org/10.1016/j.rser.2022.112752

14 Horobet, A., Tudor, C. D., Belascu, L. & Dumitrescu, D. G. The role of distinct electricity sources on pollution abatement: Evidence from a wide global panel. *Front. Environ. Sci.* **10** (2022). https://doi.org/10.3389/fenvs.2022.996515

15 Raihan, A. & Tuspekova, A. The nexus between economic growth, renewable energy use, agricultural land expansion, and carbon emissions: New insights from Peru. *Energy Nexus* **6** (2022). https://doi.org/10.1016/j.nexus.2022.100067

16 Obobisa, E. S. Achieving 1.5 °C and net-zero emissions target: The role of renewable energy and financial development. *Renew. Energy* **188**, 967-985 (2022). https://doi.org/10.1016/j.renene.2022.02.056

17 Logan, K. G., Nelson, J. D. & Hastings, A. Electric and hydrogen buses: Shifting from conventionally fuelled cars in the UK. *Transp. Res. Part D Transp. Environ.* **85** (2020). https://doi.org/10.1016/j.trd.2020.102350

18 Govindarajan, H. K. & Ganesh, L. S. Renewable energy for electricity use in India: Evidence from India's smart cities mission. *Renew. Energy Focus* **38**, 36-43 (2021). https://doi.org/10.1016/j.ref.2021.05.005

19 Cheng, Y. & Yao, X. Carbon intensity reduction assessment of renewable energy technology innovation in China: A panel data model with cross-section dependence and slope heterogeneity. *Renewable Sustainable Energy Rev* **135** (2021). https://doi.org/10.1016/j.rser.2020.110157

20 Handayani, K., Krozer, Y. & Filatova, T. From fossil fuels to renewables: An analysis of long-term scenarios considering technological learning. *Energy Policy* **127**, 134-146 (2019). https://doi.org/10.1016/j.enpol.2018.11.045

21 Cheng, C., Ren, X., Wang, Z. & Yan, C. Heterogeneous impacts of renewable energy and environmental patents on CO 2 emission - Evidence from the BRIICS. *Sci. Total Environ.* **668**, 1328-1338 (2019). https://doi.org/10.1016/j.scitotenv.2019.02.063

22 Calvillo, C. F. & Turner, K. Analysing the impacts of a large-scale EV rollout in the UK - How can we better inform environmental and climate policy? *Energy Strateg. Rev.* **30**, 11 (2020). https://doi.org/10.1016/j.esr.2020.100497

23 Hossain, M. R. *et al.* Role of energy mix and eco-innovation in achieving environmental sustainability in the USA using the dynamic ARDL approach: Accounting the supply side of the ecosystem. *Renew. Energy* **215**, 14 (2023). https://doi.org/10.1016/j.renene.2023.118925

24 Page, M. J. *et al.* The PRISMA 2020 statement: An updated guideline for reporting systematic reviews. *PLOS Medicine* **18**, e1003583 (2021). https://doi.org/10.1371/journal.pmed.1003583
